# Design, control, aerodynamic performances, and structural integrity investigations of compact ducted drone with co-axial propeller for high altitude surveillance

**DOI:** 10.1038/s41598-024-54174-x

**Published:** 2024-03-15

**Authors:** Shyam Sundar Jayakumar, Indira Prasanth Subramaniam, Beena Stanislaus Arputharaj, Senthil Kumar Solaiappan, Parvathy Rajendran, It Ee Lee, Senthil Kumar Madasamy, Raj Kumar Gnanasekaran, Arunkumar Karuppasamy, Vijayanandh Raja

**Affiliations:** 1 Department of Aeronautical Engineering, Kumaraguru College of Technology, Coimbatore, Tamil Nadu 641049 India; 2 Department of Research and Innovation, Saveetha School of Engineering, SIMATS, Chennai, Tamil Nadu 602105 India; 3 School of Aerospace Engineering, Universiti Sains Malaysia, 14300 Nibong Tebal, Penang Malaysia; 4 Faculty of Engineering, Multimedia University, 63100 Cyberjaya, Selangor D. E. Malaysia; 5 Department of Aeronautical Engineering, MLR Institute of Technology, Hyderabad, Telangana India

**Keywords:** Attitude control, Bi-copter, Composite materials, Ducted Drone, CFD, FEA, FSI, C–D duct, Thrust vectoring, UAV, Engineering, Aerospace engineering

## Abstract

Compact multi-rotor unmanned aerial vehicles (UAVs) can be operated in many challenging environmental conditions. In case the UAV requires certain considerations in designing like lightweight, efficient propulsion system and others depending upon the application, the hybrid UAV comes into play when the usual UAV types cannot be sufficient to meet the requirements. The propulsion system for the UAV was selected to be coaxial rotors because it has a high thrust-to-weight ratio and to increase the efficiency of the propulsion system, a unique propeller was proposed to achieve higher thrust. The proposed propeller was uniquely designed by analyzing various airfoil sections under different Reynolds’s number using X-Foil tool to obtain the optimum airfoil section for the propellers. Since the design with duct increases efficiency, the Hybrid UAV presented in this paper has the modified novel convergent–divergent (C–D)-based duct which is a simplified model of a conventional C–D duct. The yawing and rolling maneuverings of the UAV could be achieved by the thrust vectoring method so that the design is simpler from a structural and mechanical perspective. The use of UAVs has risen in recent years, especially compact UAVs, which can be applied for applications like surveillance, detection and inspection, and monitoring in a narrow region of space. The design of the UAV is modeled in CATIA, and its further performance enactment factors are picked from advanced computational simulations relayed bottom-up approach. The predominant computational fluid dynamics (CFD) and fluid structure interaction (FSI) investigations are imposed and optimized through Computational Analyses using Ansys Workbench 17.2, which includes analysis of structural behaviour of various alloys, CFRP and GFRP based composite materials. From the structural analysis Titanium alloy came out to be the best performing materials among the others by having lower total deformation and other parameters such as normal and equivalent stress. The dynamics control response is obtained using MATLAB Simulink. The validations are carried out on the propeller using a thrust stand for CFD and on the duct through a high-jet facility for structural outcomes to meet the expected outcome.

## Introduction

Unmanned Aerial Vehicles (UAVs) have been utilised in several fields, namely in dangerous environments or in circumstances when human presence or survival is not possible. In the past decade, the Aviation industry has made substantial progress in its capacity to operate in various environmental conditions and carry out a wide array of operations. The process of development involves the assimilation of innovative technologies, the meticulous choice of materials, the creation of structures, the integration of electrical and electronic components, and the pursuit of further study. These efforts have resulted in the development of classes that are determined by several elements, such as configurations and other pertinent aspects. An important advantage is that these vehicles can be operated remotely by a ground-based controller using radio transmission, eliminating the need for onboard operation. The design configuration can be customised to optimise accuracy for a specific mission, based on the nature of the application. UAVs are primarily classified into three basic types: fixed wing, multirotor, and rotary wing. Each of these classes has unique operating characteristics and functions. There are two prominent classifications of advanced UAVs: Hybrid UAVs and Ornithopters^1–10^.

The utilisation of UAVs in surveillance is a vast domain that offers the possibility of attaining remarkable levels of success in fulfilling diverse needs. In this situation, the utilisation of UAVs equipped with features such as vertical take-off and landing (VTOL), hovering, high manoeuvrability, and compactness can be contemplated. The main requirement for surveillance activities is to maximise the endurance of the UAV. This technology can be classified into civil or military applications. It can be employed for monitoring purposes in harsh conditions at high-altitude mountain locations in civil applications. Moreover, it can also be utilised for commercial surveillance or expediting rescue missions. Hence, it possesses the capacity to be utilised in a wide range of surveillance endeavours. Design calculations necessitate the use of both theoretical and empirical relationships. The optimisation process is improved by using different case studies, which are examined using specialised tools for design and analysis^1–5^.

Hybrid UAVs have the capacity to overcome the limits associated with vertical takeoff and landing (VTOL) and other constraints. The design can be customised to fulfil precise specifications, while further intricacies can be resolved by further optimisation. The utilisation of composite materials for weight reduction is extensively adopted due to its exceptional strength-to-weight ratio and superior characteristics in comparison to other currently available materials. The incorporation of a coaxial arrangement in a Monocopter design enhances its resilience; nevertheless, it requires a greater power input to provide the same amount of thrust as an isolated form. The combined thrust produced by a coaxial design exceeds that of a standalone configuration, hence enhancing the capacity to propel the current model being examined. Utilising a duct in a propulsion system has the capacity to improve efficiency by minimising tip losses. The literature review focused on many factors including the design configuration, propulsion systems, control mechanisms, and several other areas^5–10^.

### Literature survey

To proceed the research with proper and necessary methodology, design and computational parameters, a detailed literature survey was performed. From the performed literature review, numerous data about the computational methods imposed and information about various UAVs were obtained and they are classified according to their design, computational fluid dynamics (CFD), finite element analysis (FEA), deployment, and components used, and are depicted in Table 1.

**Table 1:**
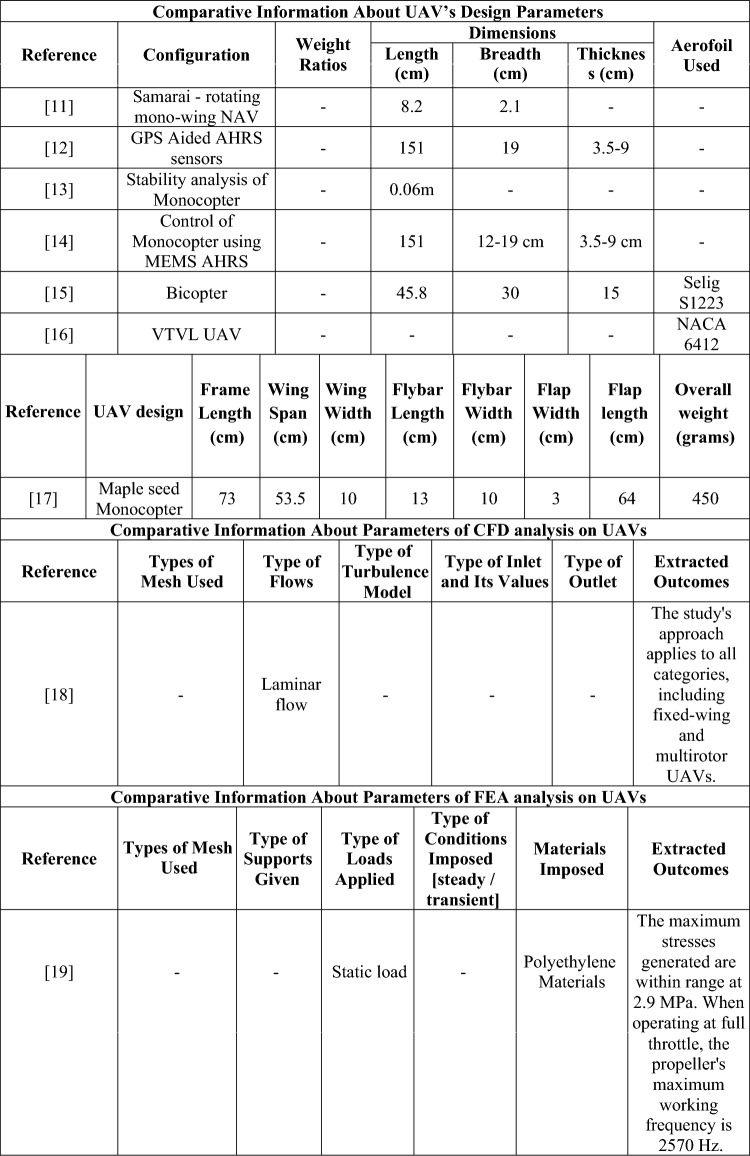

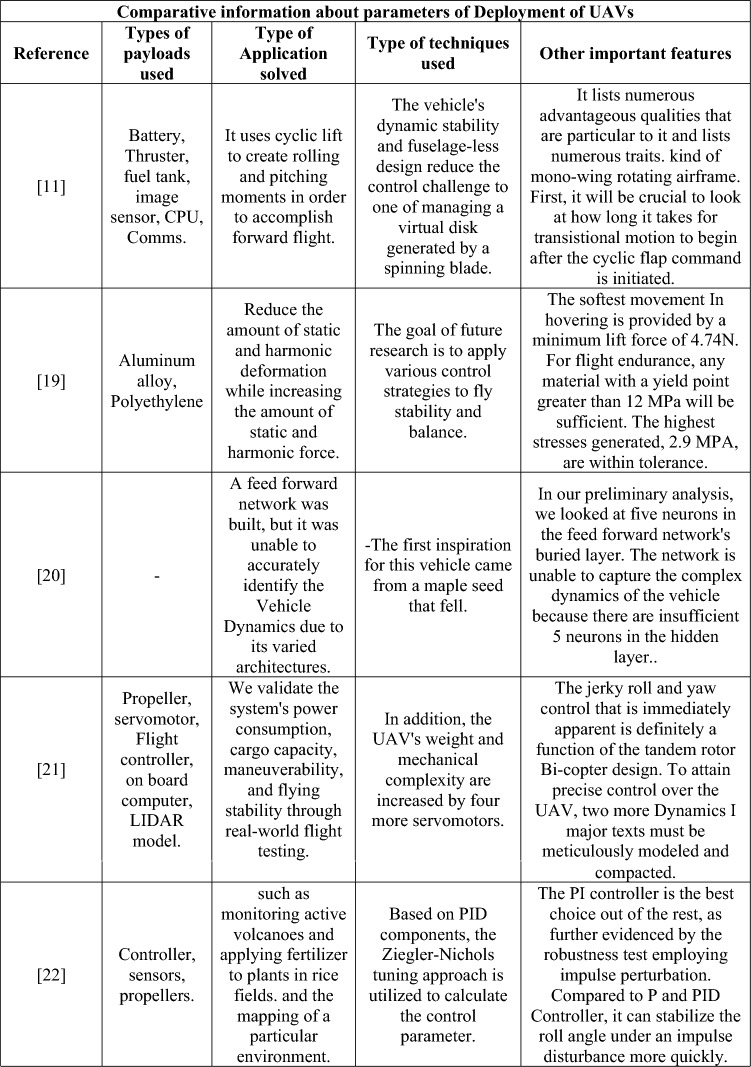

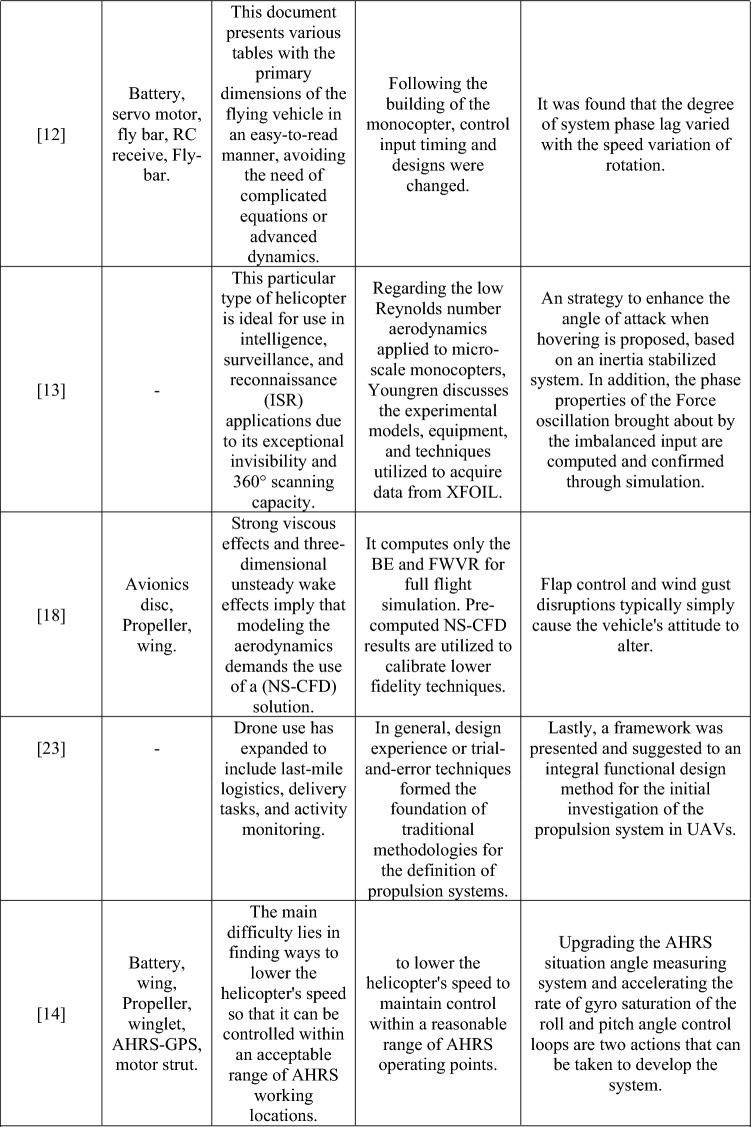

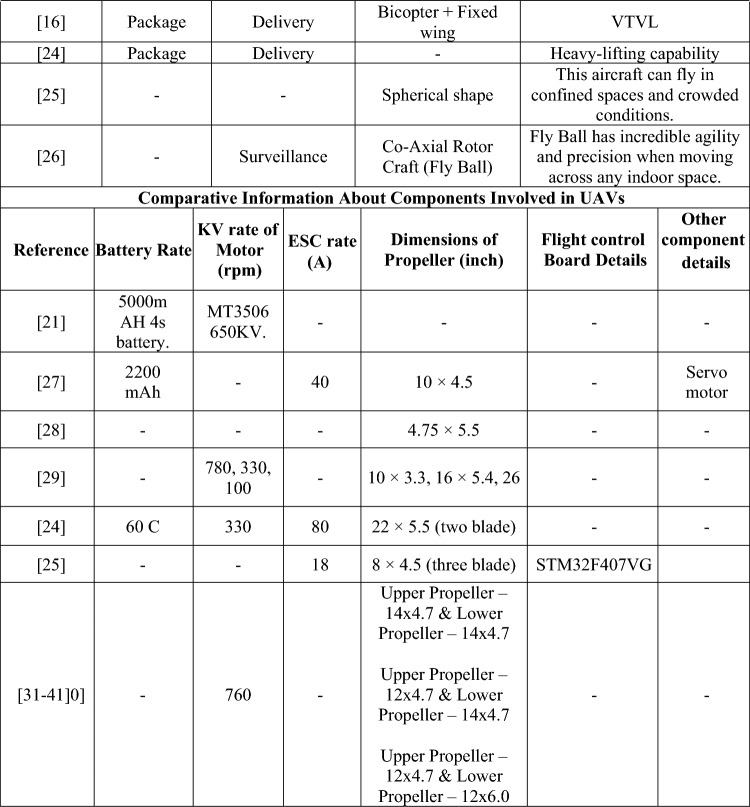
Comprehensive information about complete investigation data on UAV.

Table 1 provides the collected data from the performed literature review which was used for the development of the research. The identification of research gaps is determined through the fieldwork activities outlined as follows: Firstly, the utilization of totally enlarged outer casing-based ducted drones predominantly resulted in the imposition of higher pressure and skin friction drags in a suitable manner. Henceforth, less drag developing based duct’s outer casing was failed to incorporate in ducted drones. Secondly, the primary source of thrust for drones equipped with ducts was a single propeller. Due to the significant influence of the beginning condition on the exit induced velocity of the ducts, it is possible that the thrust developed by a single propeller-based propulsion system may not meet the needed threshold. Thirdly, it is worth noting that the majority of ducted drones now on the market are equipped with imposed conventional propellers. The traditional propeller may not be suitable for all types of manoeuvres. The utilization of a distinctive propeller design with significant lifting capabilities is essential for ducted drones, as opposed to the traditional propeller configuration. Fourthly, the ducted casings were produced using typical lightweight alloys and composites based on glass fibre. The structural collapse of ducted drones during transition manoeuvring may occur due to the application of modest weight and load-bearing capacity of the material.

The effective design and optimization of a small Hybrid UAV can be accomplished by leveraging present computing technologies, specifically by applying numerical analytic techniques. The preliminary stage of the design process entails the estimation of the aircraft’s weight. Subsequently, the design process entails the development of distinct components, namely the propulsion system encompassing the propeller and motor, the mechanisms essential for accomplishing mission objectives such as a camera and holder, the fuselage with a specific focus on the duct, and the thrust vectoring mechanism comprising of deflector plates. The model under consideration is created using the computer-aided design software called CATIA. The model is subjected to testing utilizing the operational ambient conditions within the simulation programme, Ansys Workbench 17.2. A numerical simulation is performed, and the model is subsequently optimized using the acquired data. The individual evaluation of the components of the UAV is conducted in order to improve optimization. Following this, the entire model will be built and subjected to computational testing. The determination of the required components for the functioning of the UAV is dependent on the final design. During the design phase, it is customary to preselect specific components, particularly the major components that are important for the model. After finalizing the general design, the additional components required for the model are subsequently chosen.

To conduct the simulation of the altitude control and reaction of the UAV, The MATLAB Simulink is employed, which is contingent upon the input. The main focus of this study centers on translational motion, as the simulation is carried out in two distinct phases: the Hovering stage and the VTOL stage. This examination provides a more thorough understanding of the control system utilized in UAVs, which is dependent on the particular model and its propulsion components. The propeller employed in the UAV is subjected to experimental verification through the utilization of a thrust stand. The model is subsequently validated through a comparative analysis of the theoretical, numerical, and experimental results.

### Mission profile

**Figure 1:**
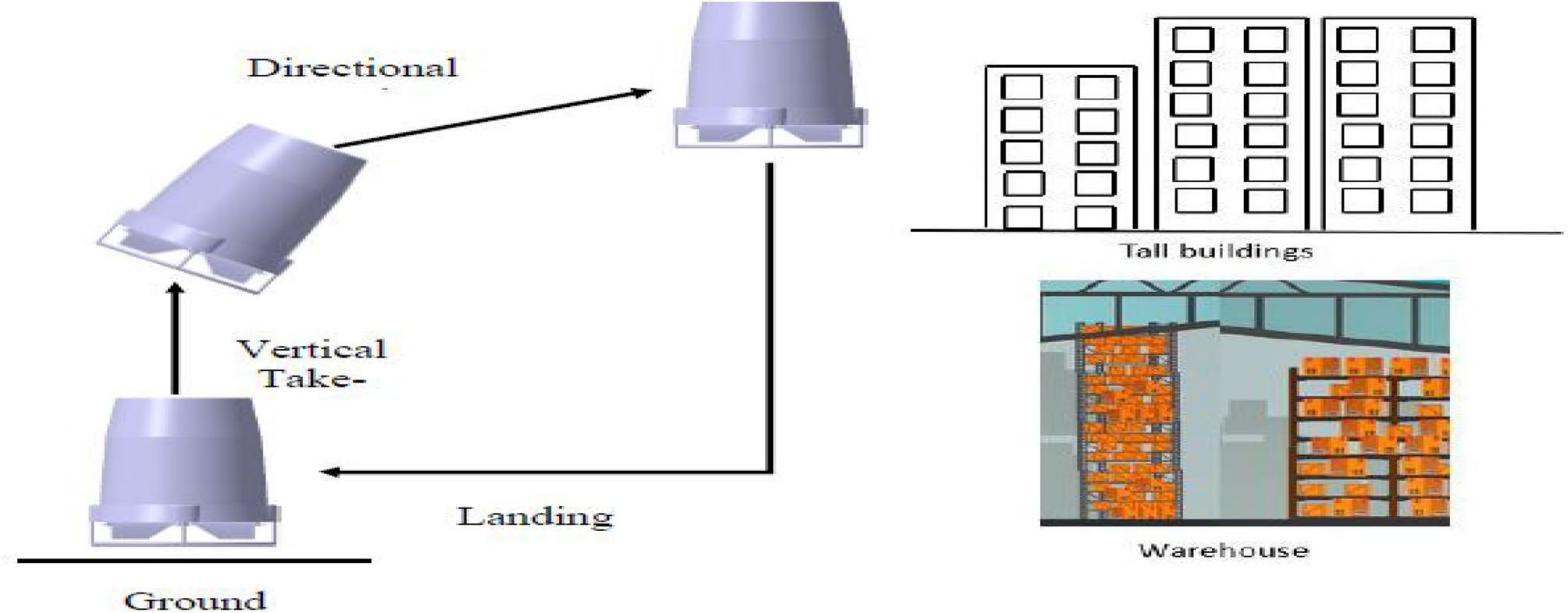
Pictorial representation of mission profile of the Mono-copter and possible places of its application.

**Figure 2:**
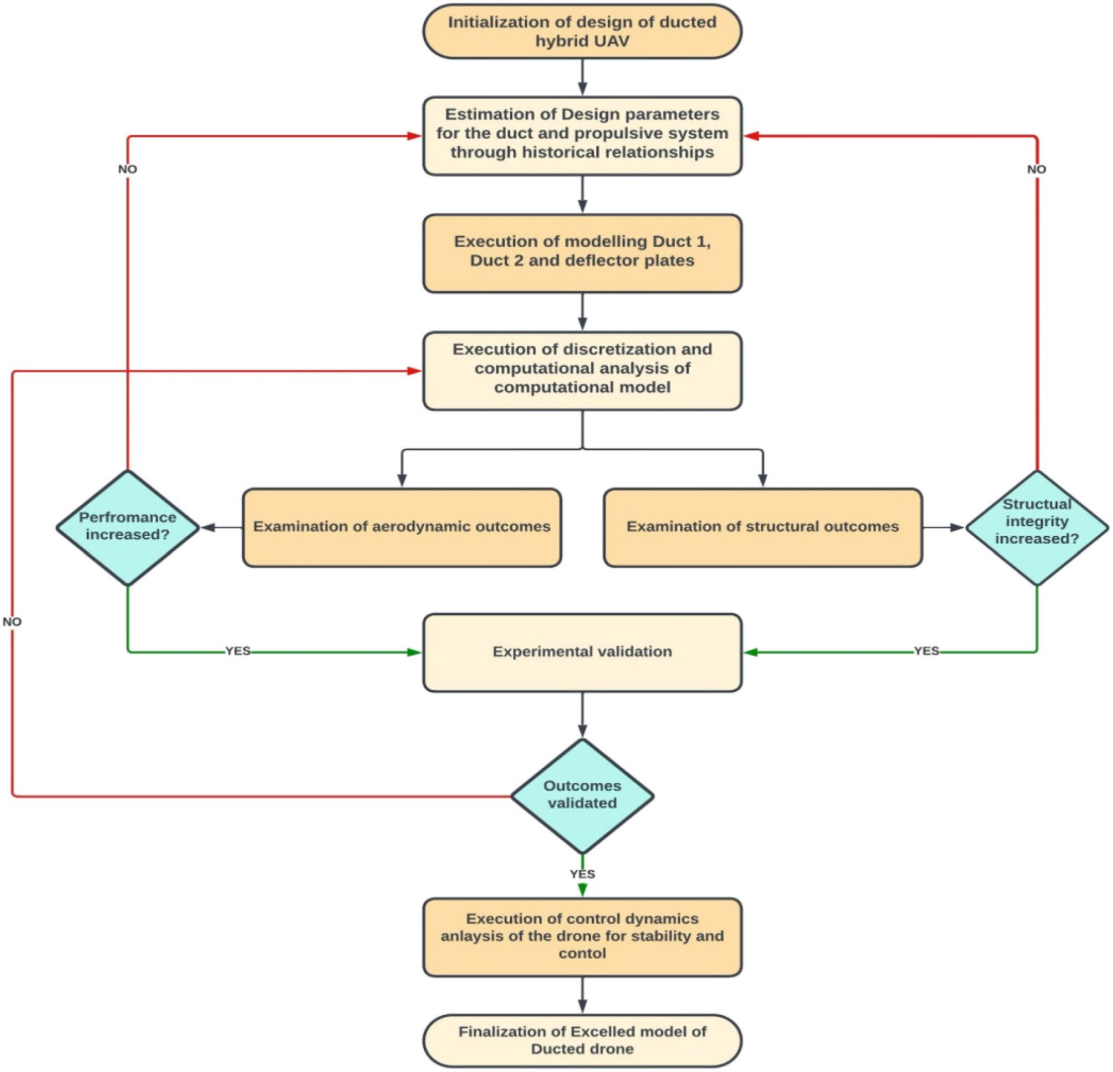
Methodology work flow of the research.

The proposed mission profile of this advanced multi-rotor and its application are represented in Figs. 1 and 2 clearly depicts the methodology flow followed for the research.

## Design of hybrid UAV

The design phase of the Hybrid UAV consists of weight estimations, design parameters calculation, the outline of the model and finally the components.

### Weight estimation

The estimation phase involves the primary components that are required for the design of the UAV, so that the weight of those components will be added and an average of overall weight from the historical data’s contributing to calculate the overall weight the UAV to be designed^30–40^.

#### Payload weight estimation

The process began with the payload weight estimation in which various camera data were collected from the historical references and Table 2 depicts the type, weight, dimension and their application.

**Table 2:** Comprehensive information of relevant Payload data ^5–10^.

S. No.	Camera	Type	Weight (grams)	Dimension (mm)	Application
1	FLIR GF620	Thermal camera	–	–	Methane gas leak detection
2	XIMEAMQ013MG-E2	Imaging camera	26	26 × 26 × 26	autonomous monitoring, inspection, and surveillance of buildings, (maintenance in industrial plants)
3	UI-1221LE Rev. 2	Imaging Camera	12	36.0 × 36.0 × 20.2	vessel inspection
4	X5S DJI Zenmuse	Imaging Camera	499	139.7 × 132.1 × 190.5	Inspections of construction stormwater practices
5	Canon PowerShot SX220 HS	Imaging Camera	215	105.7 × 59.3 × 33.2	Remote building inspection and monitoring
6	Stereo camera	–	–	–	
7	Stereo camera	Imaging Camera	80.5 (Enclosed) 28.5 (unenclosed)	100 × 30 × 35	Indoor chimney inspection
8	FPV camera	Video recording and Imaging	18	50 × 30 × 10	Video recording and imaging
9	FPV camera	Video recording and Imaging	20	70 × 50 × 40	Video recording and imaging
10	Sport camera	Video recording and Imaging	40	100 × 70 × 65	Video recording and imaging

From Table 2, the appropriate camera required for the mission was comparatively, the sport camera. It is more fit to execute this proposed mission thus the same camera was picked.

1 $${\text{W}}_{{{\text{Payload}}}} = {\text{W}}_{{{\text{Primary}}}} + {\text{W}}_{{{\text{Secondary}}}}$$

From the collected data in Table 2, ${\text{W}}_{{{\text{Primary}}}} = {\text{W}}_{{\text{Sport Camera}}} \Rightarrow 40{\text{ grams}};{ }$ andso ${\text{W}}_{{{\text{Secondary}}}} = {\text{W}}_{{\text{Rotating devices}}} + {\text{W}}_{{\text{Holding devices}}} \Rightarrow 30 + 25 \Rightarrow 55{\text{ grams}}.$ All of these data are substituted in Eq. (1)^30–40^, and thereafter the payload weight (${\text{W}}_{{{\text{Payload}}}}$) was estimated. So, this proposed UAV’s payload was estimated to be, ${\text{W}}_{{{\text{Payload}}}} = {\text{W}}_{{{\text{Primary}}}} + {\text{W}}_{{{\text{Secondary}}}} \Rightarrow 40 + 55 = 100{\text{ grams}}$. With this the overall weight of the UAV can be estimated.

#### Estimation of Overall Drone Weight

**Figure 3:**
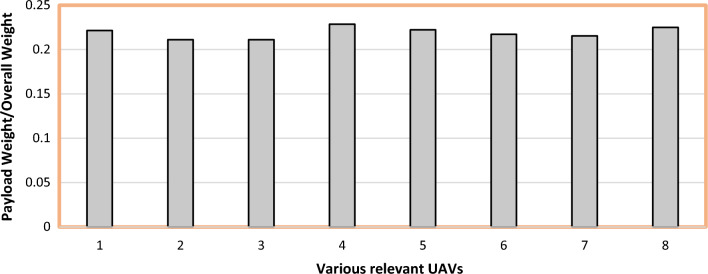
Comprehensive study on historical relationship of UAVs and its weight.

The relation between the payload weight and the overall weight of UAV was derived from the historical references, which is represented graphically in Fig. 3. With the help of derived relation in Eq. (2) which gives the relation between the payload and takeoff weight, and with the payload weight obtained from Eq. (1) we estimated the overall take-off weight (${{\text{W}}}_{{\text{Take}}-{\text{Off}}}$) of the UAV to be 460 g^30–40^. This estimation is crucial for the estimating the thrust requirement for the UAV and its propeller design.

2 $$\frac{{{\text{W}}_{{{\text{Payload}}}} }}{{{\text{W}}_{{\text{Take - Off}}} }} = 0.21814$$

$${\text{W}}_{{\text{Take - Off}}} = \frac{{{\text{W}}_{{{\text{Payload}}}} }}{0.21814} \Rightarrow \frac{0.1}{{0.21814}} = 460\;{\text{g}}$$

### Propeller design

This section discusses about the calculations and determination of diameter, pitch angle, airfoil selection and other design parameters for the design of the propellers. The design of the propeller is purely our own and to be produced based on our requirement^30–40^.

#### Estimation of diameter of the co-axial propeller

Thrust to weight ratio is one of the crucial parameters for any multi rotor UAV^30–40^. The relation between the thrust produced by the rotors and the overall weight of the UAV is shown in Eq. (3) as follows:

3 $${\text{Thrust to Weight Ratio}} = { }\frac{{\text{Thrust*Number of Rotor}}}{{\text{Weight of the multi rotor UAV }}}$$

In this work, thrust to weight ratio is assumed as 2 that is comparatively higher value. Since the working environment of this proposed application is complicated in terms of high gust production, the higher value-based thrust to weight ratio has been chosen^30–40^ So that the drone is capable of maneuvering in any unexpected harsh conditions. Thus,

$${\text{Thrust requirement of the single propeller}} = { }\frac{{2{\text{ x }}460}}{2} \Rightarrow 460{\text{ grams}}$$

The calculated thrust gave a lead for the requirements for the propulsive device’s design parameters.

From the trade-off analysis, the maximum VTOL speed of this recommended UAV was fixed as 20 m/s^30–40^. Henceforth the exit velocity is ${\text{ V}}_{{\text{e}}} = 20{\text{ m}}/{\text{s}}$, measured average wind velocity at the working environment is ${\text{ V}}_{{\text{a}}} = 4{\text{ m}}/{\text{s}}$.

4 $${\text{T}} = 0.5 \times {\uprho } \times {\text{A}} \times \left[ {\left( {{\text{V}}_{{\text{e}}} } \right)^{2} - \left( {{\text{V}}_{{\text{a}}} } \right)^{2} } \right]$$

By imposing these acquired values such as thrust required, exit and average velocity and density in Eq. (4), the area of the rotor was calculated. With the acquired area, the diameter of the propeller was estimated as 6.1575 inches^30–40^.

#### Estimation of maximum requirement of power

Power requirement directly influences the flight time and the endurance of the UAV. Thus, the maximum amount of power required for the UAV to complete its mission was obtained from Eq. (5) by imposing the required parameters such as obtained area of the rotor, maximum velocity of the UAV and its operating altitude for density^30–40^.

5 $${\text{Power}} = \frac{1}{2} \times {\text{T}} \times {\text{V}}_{{{\text{Max}}}} \times { }\left[ {\left( {\frac{{\text{T}}}{{{\text{A}} \times {\text{V}}_{{{\text{Max}}}}^{{2{ }}} \times { }{\raise0.7ex\hbox{${\uprho }$} \!\mathord{\left/ {\vphantom {{\uprho } 2}}\right.\kern-0pt} \!\lower0.7ex\hbox{$2$}}}} + 1} \right)^{\frac{1}{2}} + { }1} \right]$$

The power required of 107.9307 Watts directly contributes to the estimation of the design parameters of the propulsive devices.

#### Estimation of propeller’s pitch

To estimate the propeller pitch, the maximum rpm and the forward speed of the propeller are required. Equations (6) and (7) express the relationships of power and thrust of the propeller. With the help of these two equations, the working rpm of this developed propeller was estimated. Henceforth, the pitch of the same propeller is determined with the help of Eq. (8) by imposing the required parameters such as diameter of the rotor and power required^30–40^.

6 $${\text{P}} = {\text{k}} \times {\text{R}}^{3} \times {\text{D}}^{4} \times {\text{p}}$$

7 $${\text{T}} = 4.392399 \times { }10^{ - 8} \times {\text{RPM}} \times \frac{{({\text{d}}^{3.5} )}}{{\sqrt {{\text{pitch}}} }} \times \left[ {4.23333 \times 10^{ - 4} \times {\text{RPM}} \times {\text{Pitch}} - {\text{V}}_{{{\text{Forward}}}} } \right]$$

The estimated RPM from the calculations was 17,080.

8 $${\text{Main}}\;{\text{Rotors}}\;{\text{Pitch}} = \frac{{{\text{Induced}}\;{\text{Velocity}}\;{\text{in}}\frac{{{\text{inch}}}}{{\text{s}}}}}{{{\text{Revoution}}\;{\text{Per}}\;{\text{Second }}}} = {\text{inch}}/{\text{revolution}}$$

The pitch of the rotor was estimated as 5.7 inch/rev which can produce the required thrust for all maneuvers.

#### Estimation of C_L_ and Induced velocity at Hovering

Hovering is a significant maneuver for any type of UAV and the aerodynamic parameters acting on the UAV during hover is the maximum at any point of its whole mission profile. The lift required to hover the UAV from ground is its own weight^30–40^. Thus, the lift required for single propeller to hover the UAV is:

$${\text{Lift requirement of the single propeller}} = { }\frac{0.46}{2} \Rightarrow 2.2563{\text{ N}}$$

With the conventional lift equation and the lift obtained the relation was simplified in Eq. (9) and it is as follows:

9 $$\left[ {{\text{V}}_{{\text{Rotational Speed at Hovering}}} } \right]^{2} {\text{ x C}}_{{{\text{L}} - {\text{Hovering}}}} = 191.77$$

By assuming Thrust = Weight the Thrust equation can be written in the form as in Eq. (10)^30–40^. Now, by applying the area of the rotor and the density at sea level ($\uprho$), the velocity at which the rotor rotates at hovering is finalized as:

10 $${\text{W}} = 0.5 \times\uprho \times {\text{A}} \times \left[ {\left( {{\text{V}}_{{{\text{Rotational}}\;{\text{Speed}}\;{\text{at}}\;{\text{Hovering}}}} } \right)^{2} - \left( {{\text{V}}_{{\text{a}}} } \right)^{2} } \right]$$

The rotational speed at hovering maneuver is estimated as 14.4255 m/s.

From Eq. (9), the coefficient of lift at hovering (${\text{C}}_{{{\text{L}} - {\text{Hovering}}}}$) was determined.

$${\text{C}}_{{{\text{L}} - {\text{Hovering}}}} = { }\frac{191.77}{{\left( {14.4255 \times 14.4255{ }} \right)}} = 0.92155$$

The acquired C_L-Hovering_ contributes to the airfoil selection for the propeller which can produce the required coefficient of lift with a minimum drag possible.

#### Estimation of Pitch angle and Chord of the Propeller

Pitch angle is the angle between the rotor’s plane of rotation and the chord of the rotor. Proper pitch angle produces the optimum lift to drag ratio (L/D) for the rotor. The relation to find the pitch angle is depicted below in Eq. (11)^30–40^.

11 $$\uptheta = {\text{arctangent}} \left( {\frac{{\text{P}}}{{2 \times\uppi \times {\text{r}}}}} \right)$$

With the obtained pitch angle, chord angle can be determined by using the Eq. (12)^30–40^ and can be used for the propeller design.

12 $${\text{b}} = \frac{{8 \times\uppi \times \left( {\frac{{\sin \left( {\uptheta } \right) \times \left( {\tan \left( {\uptheta } \right) - \frac{1}{1.2} \times \tan \left( {\uptheta } \right)} \right)}}{{\left( {1 + { }\frac{1}{1.2} \times \tan \left( {\uptheta } \right)} \right)}}} \right) \times {\text{r}}}}{{{\text{n}} \times {\text{C}}_{{\text{L}}} }}$$

As for the propeller having a varying cross section, with the above relations the Pitch angle and chord length of the propeller is determined for 10 different locations of the propeller and are represented in Table 3. With this calculated data, our own propeller was designed for our requirement^30–40^.

**Table 3:** Design Details of MRUAV’s Propeller.

Sl. No.	Pitch angle (θ) (degree)	Location (inches)	chord length (inches)
1	71.7099	0.307087	0.55551181
2	56.5332	0.6141732	0.76023622
3	45.2423	0.92086614	0.79488189
4	37.1027	1.2279528	0.7622047
5	31.1779	1.5350394	0.70905512
6	26.7594	1.842126	0.65314961
7	23.3745	2.1492126	0.6011811
8	20.7159	2.4559055	0.55433071
9	18.5808	2.7629921	0.51299213
10	16.8331	3.0622047	0.476378

The estimation of Reynolds Number is unavoidable for the development and investigation of propeller. Thus, the conventional relationship of the Reynolds Number is systematically expressed in Eq. (13) with the available data of density, velocity and the appropriate length, the operating Reynolds’ number was estimated as follows:^30–40^.

13 $${\text{Re}} = \frac{{\uprho \times {\text{v}}_{f} \times l}}{\upmu }$$

$${\text{Re}} = { }\frac{{1.2256{\text{ x }}20{\text{ x }}0.1559587}}{0.000018} \Rightarrow {\text{Re}} = 212381.1$$

The obtained Reynolds’s number shows the operating range of the UAV which is very crucial for aerofoil selection, so that the propulsion system produces the estimated required thrust for the UAV.

#### Selection of aerofoil

**Figure 4:**
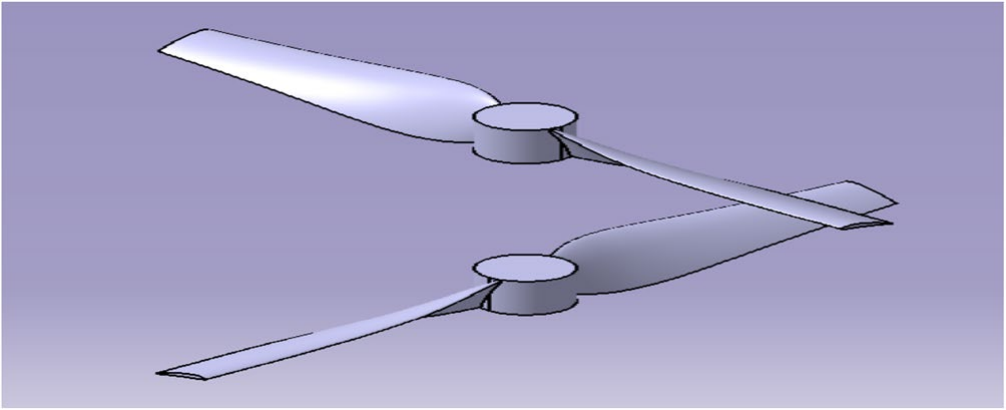
A systematic view of contra-rotating propellers of the drone.

The Aerofoil was selected based on the coefficient of lift and the minimum drag. Based on the requirement, NACA 6412 came out to be the best performer from analyzing various aerofoils using XFOIL tool, and was selected for the propeller design^30–40^.The distance between the two coaxially powered propellers will be efficient at 50 mm. Figure 4 represents the CAD model of the co-axial Propeller’s designed using CATIA.

### Design of duct configuration

Based on our study we propose two Duct designs in order to minimize the tip losses and also various other factors leading to reduction in efficiency of the propeller.

#### Duct design configuration-1

This design is similar to a C–D Nozzle like configuration in which it has a convergent and a divergent section. The propulsion system is placed above the duct in this configuration Duct’s inlet diameter is concluded from Propeller’s Diameter with a 10% increase, in order to have clearance between propeller and the duct. The total inlet diameter of the duct is calculated as per Eq. (14)^30–40^.

14 $${\text{Duct inlet Diameter}} = {\text{Propeller Diameter }} + { }\left( {10{\text{\% of Propeller Diameter}}} \right)$$

With the inclusive of calculated propeller design data, the duct inlet diameter and it’s area were calculated as follows^30–40^: Duct inlet Diameter = 155.9587 + (15.59587) mm ⇒171.55457 mm and ${\text{Duct inlet Area }}\left( {{\text{A}}_{{\text{i}}} } \right) = {\pi r}^{2} = 23124.3262{\text{ mm}}^{2}$.

15 $$\frac{{A_{i} }}{{A_{t} }} = \left( {\frac{\gamma + 1}{2}} \right)^{{\frac{{ - \left( {\gamma + 1} \right)}}{{2\left( {\gamma - 1} \right)}}}} \frac{{\left( {1 + \frac{\gamma - 1}{2}M^{2} } \right)}}{M}^{{\frac{{\left( {\gamma + 1} \right)}}{{2\left( {\gamma - 1} \right)}}}}$$

Using the Area-Mach relation in Eq. (15) the duct’s throat area was estimated as, $A_{t} = \frac{{A_{i} }}{9.945} \Rightarrow A_{t} = 2325.2147 {\text{mm}}^{2}$ by imposing the values of γ = 1.4 (ratio of specific heats); M = 0.0583 (operating Mach number); $\frac{{{\text{A}}_{{\text{i}}} }}{{{\text{A}}_{{\text{t}}} }} = 9.945$ (Ratio of inlet and throat area); Duct’s Divergence section is also calculated based on propeller diameter with an increase in 20%, since dealing with low Mach number^30–40^.

16 $${\text{Duct Outlet Diameter }} = {\text{ Propeller Diameter}} + \left( {20{\text{\% of Propeller Diameter}}} \right)$$

**Figure 5:**
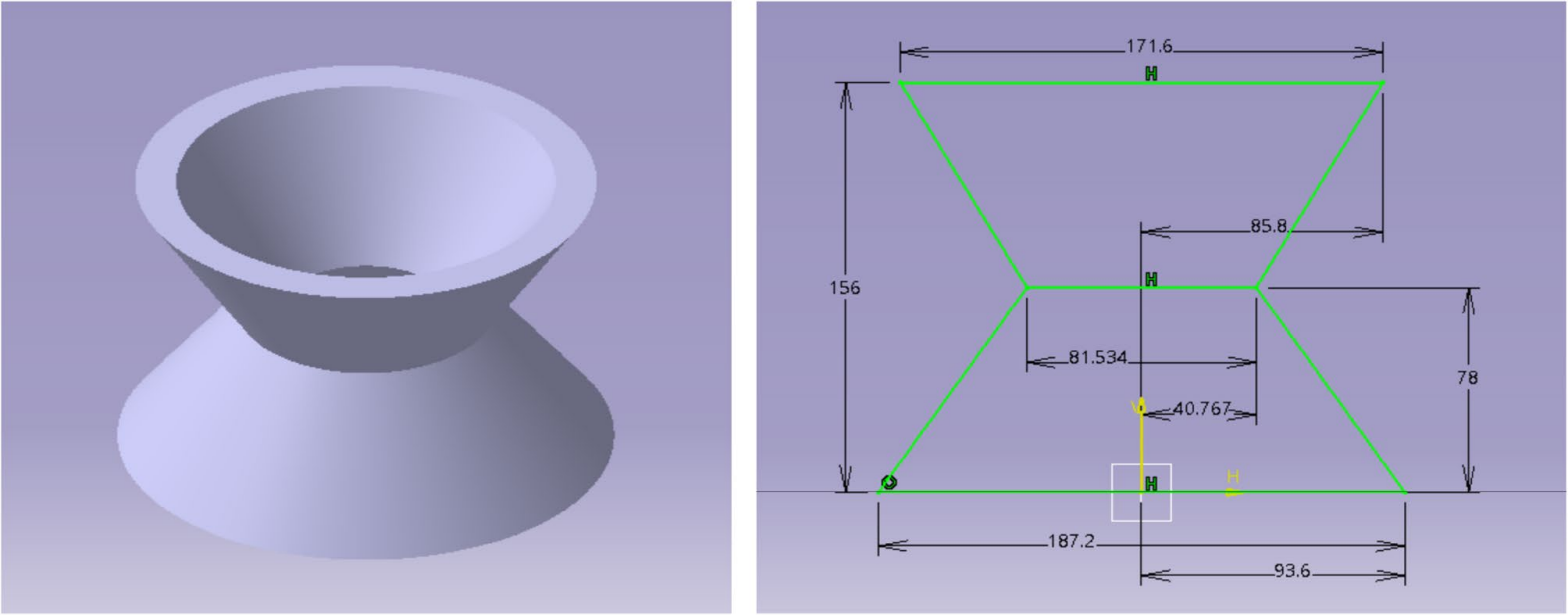
A typical isometric view of the duct Configuration 1 (all dimensions are in mm).

Thus, Duct’s Outlet Diameter = 155.9587 + (31.19174) mm ⇒ 187.15044 mm was calculated using Eq. (16); and so, duct’s outlet area is 27,519.8593 mm^2^. This work won’t be needed to create such a high Mach number at the outlet of the duct and hence the throat area is increased in such a way to obtain the required velocity. So, the angle between the throat and inlet is fixed to 120˚.Applying this, we get, ${\text{Duct Outlet Diameter}} = 81.534{\text{ mm and }}A_{t} = 5223.2661 {\text{mm}}^{2}$ these design parameters were estimated to produce the expected exit velocity. The thickness of the Duct is 10% of overall length of the duct design, which was considered from empirical datum. Hence the overall length of the duct is 156 mm and thickness of the duct is 15.6 mm. With the obtained data for duct configuration 1, the CAD model was designed, and it is portrayed in Fig. 5.

#### Duct design configuration-2

**Figure 6:**
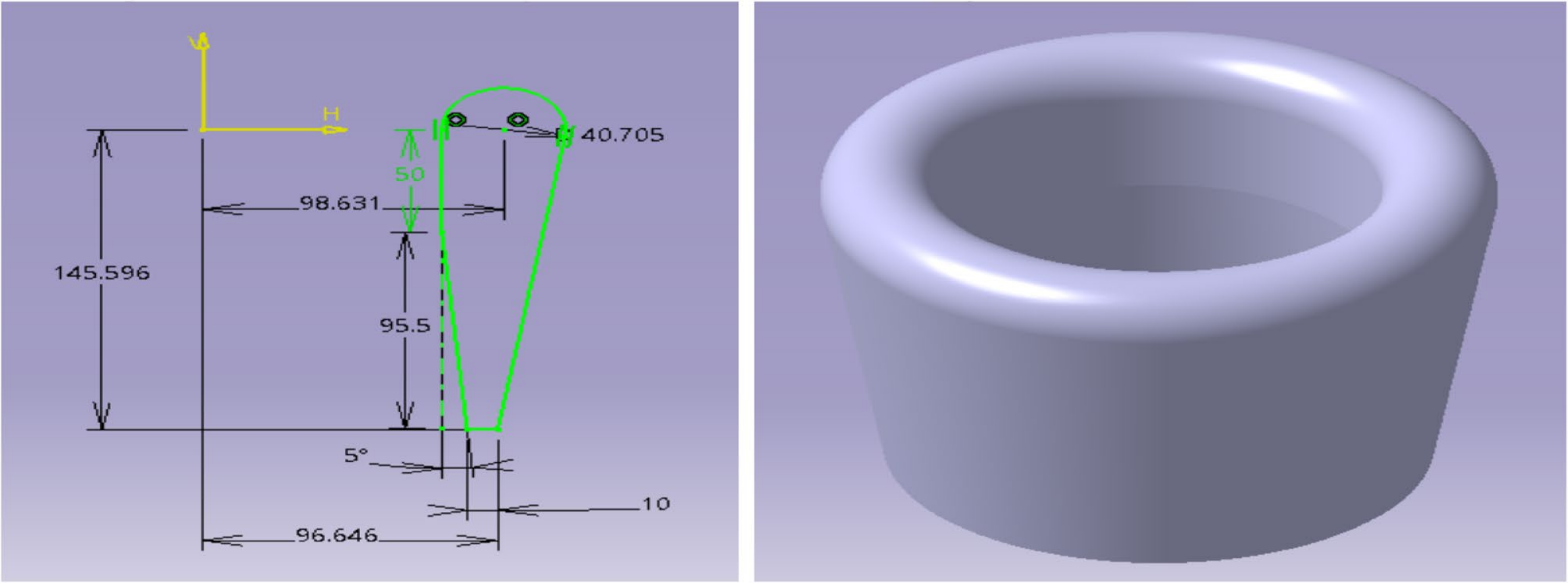
A typical isometric view of duct configuration 2 (all dimensions are in mm).

This design configuration is similar to the first design but varies in few aspects. The main difference includes the propulsion system placement and a curvy convergent section. Based on the fore-mentioned proposed method^1^ the duct can be designed based on the below parameters,${\updelta }_{{{\text{tip}}}} = 0.1{\text{\% D}}_{{\text{t}}}$, ${\text{r}}_{{{\text{lip}}}} = 13{\text{\% D}}_{{\text{t}}}$, $\uptheta _{{\text{d}}} = 10^\circ$, ${\text{L}}_{{\text{d}}} = \left( {50{\text{\% }} - 72{\text{\% }}} \right){\text{D}}_{{\text{t}}}$. Based on the propeller Diameter i.e., ${\text{D}}_{{\text{t}}} = 156.557{\text{ mm}}$; the Duct 2 Design Parameters were estimated. The significant values for the design are: ${\updelta }_{{{\text{tip}}}}$ = 0.1566 mm, ${\text{r}}_{{{\text{lip}}}}$ 20.3524 mm, ${\uptheta }_{{\text{d}}}$ = 5° and ${\text{L}}_{{\text{d}}}$ = 95.4998 mm. With these design parameters for duct 2, a computational model was designed, and it is illustrated in Fig. 6^30–40^.

### Thrust vectoring deflectors

The main maneuver control system of the UAV is this deflector, which provides the required force to achieve its maneuvering movement in case of yawing and rolling. The deflector’s height is configured based on duct design parameters. The analysis results recorded in Table 4 provides the optimizations to be done for the deflector design^30–40^.

**Table 4:** Deflector design parameters.

Surface Area of the deflector	0.012 m^2^
Height of the deflector	39 mm
Length of the deflector	184 mm
Aerofoil	NACA 0012

**Figure 7:**
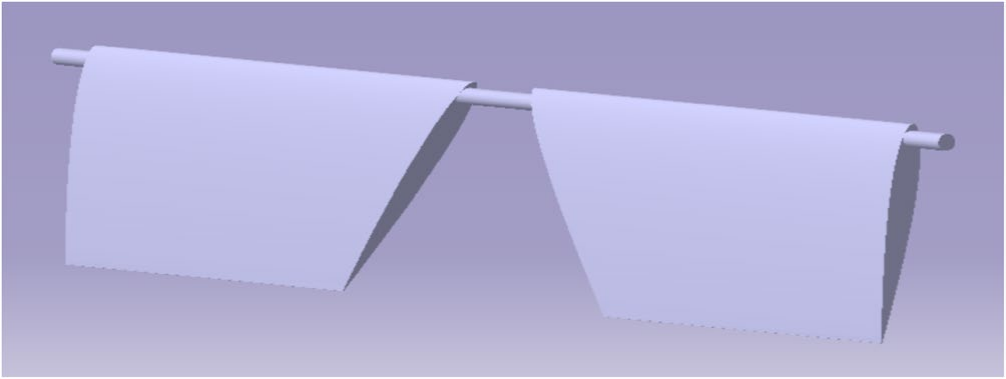
An emblematic view of the deflecting plate.

**Figure 8:**
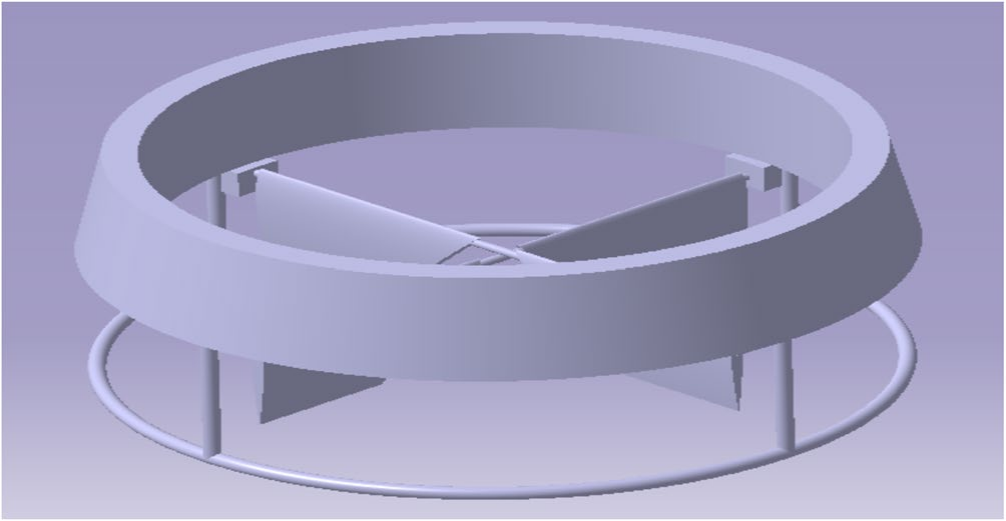
A typical isometric view deflector’s alignment in the Bottom part of model 2.

From the data in Table 4, it is clear that the aerofoil selected has a symmetrical cross section to create forces on both sides of its surfaces. Figure 6 has a typical view of the deflector plates. Figure 7 depict the deflector plates and Fig. 8 depicts the plates attached to the bottom part of duct 2.

### UAV CAD models

**Figure 9:**
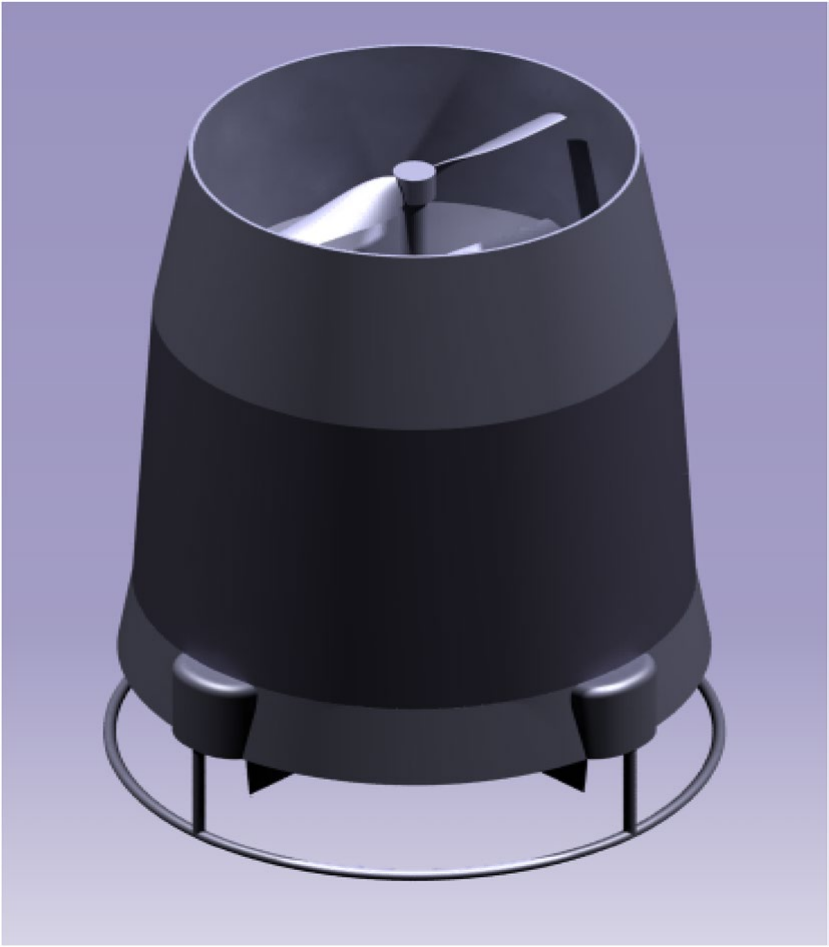
An emblematic view of UAV Design Configuration 1.

**Figure 10:**
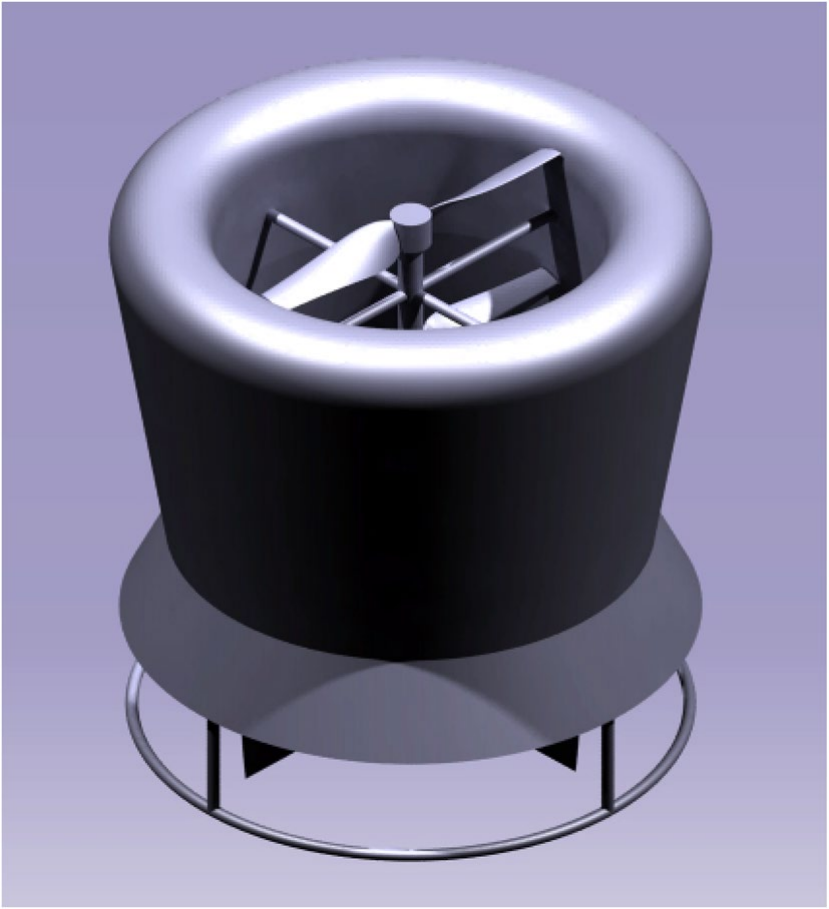
An emblematic view of UAV Design Configuration 2.

A complete design model of the UAV was assembled with all the components using CATIA V5. Figure 9 comprises of the UAV design 1 with the duct design 1 and design 2 of the UAV is displayed in Fig. 10 with the duct design 2. These were the final CAD models used to analyze aerodynamic performance analysis^30–40^.

### Components

The primary design components are selected earlier which provided the base for the design of the UAV. The secondary components represent the performance of the UAV. Like the primary components these play a crucial role in the effectiveness of the UAV. So, they are selected as follows.

An adequate amount of power is required to complete the assigned mission for the UAV. The battery needs to have an appropriate discharge rate, capacity and must be suitable within the design constraints of the UAV. From Table 5 we selected the 3S battery which satisfied our required parameters^30–40^.

**Table 5:** Comparison of Batteries.

Battery Cell type	Capacity (mAh)	Volt (V)	Discharge rate (C)	Net weight (grams)	Dimension (mm)
2S	1550	7.4	45	95	90.5 × 30.5 × 16.5
3S	1550	11.1	75	136	72 × 36 × 27
4S	1550	14.8	75	176	72 × 36 × 35
6S	1500	22.2	40	252	71 × 36 × 43

The motor selection is determined based on the major component chosen, with the following parameters being utilized for the selection process. The overall Weight (W_o_) is calculated as 460 g, the thrust to weight ratio is assumed as 2, the thrust required by single motor and propeller is determined as 460 g, the average wind velocity (V_a_) is found as 4 m/s; and Propeller design found as 6.16″ × 5.7″. The required RPM can be calculated using the below formula in Eq. (17) with the help of obtained pitch, diameter of the propeller and the thrust required as follows:

17 $$\begin{aligned} {\text{F}} & = 1.225 \times { }\frac{{{\uppi } \times \left( {0.0254 \times {\text{d}}} \right)^{2} }}{4} \times \left[ {\left( {{\text{RPM}}_{{{\text{prop}}}} \times { }0.0254 \times {\text{pitch}} \times \frac{{1{\text{ min}}}}{{60{\text{ sec}}}}} \right)^{2} } \right.{ } \\ & \quad \left. { - \left( {{\text{RPM}}_{{{\text{prop}}}} \times { }0.0254 \times {\text{pitch}} \times \frac{{1\;{\text{min}}}}{{60\;{\text{sec}}}}} \right){\text{V}}_{0} } \right] \times \left( {\frac{{\text{d}}}{{3.29546{\text{*pitch}}}}} \right)^{1.5} \\ \end{aligned}$$

**Figure 11:**
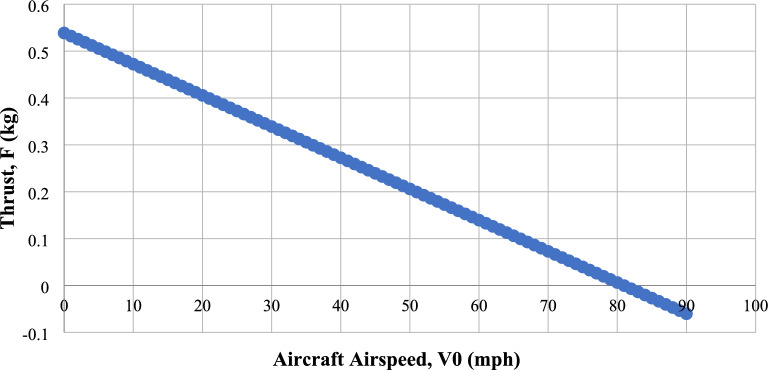
Comprehensive study on thrust production with respect to UAV’s airspeed.

By changing the RPM in the above relation, we can attain the amount of thrust required for our UAV. As a result, the optimum RPM is obtained in relation to Thrust and UAV Airspeed. Figure 11 depicts the variation of Airspeed of the UAV and thrust on a constant RPM^30–40^.The required RPM is 15,000. Since the UAVs won’t be operated in maximum RPM available, the RPM is approximated to maximum of 16,000. With this, the required kV rating of the motor was calculated as follows:${\text{kV}} = \frac{{{\text{RPM}}}}{{{\text{Battery}}\;{\text{Volts}}}} \Rightarrow \frac{16000}{{11.1}} \Rightarrow 1441.44.$ The kV of the motor can be selected between 1500 and 1700. Table 6 shows all the required parameters for the selection of motor^30–40^.

**Table 6:** Technical specification of Motor.

Specification	Chosen data	Specification	Chosen data
kV	1700 kV	No of cells	3–6S
Weight	33.4 g	Diameter	27.5 mm
Idle current (10 V)	1.2 A	Length	33.2 mm
Internal Resistance	72 m ohm		

The selection of the electronic speed controller (ESC) was finalized using the relation below and Table 7 depicts the specifications of the selected ESC. ESC is used to control the voltage supply for the motors to manipulate the RPM of the motors.

**Table 7:** ESC Specifications.

Specification	Chosen Data	Specification	Chosen Data
Model	20ABLHeli-S OPTO ESC	Peak Current(10 s)	25A
Suitable Li-Po Batteries	2–4S	Dimensions (mm) L × W × H	27 × 12 × 5
Board size	27 × 12 mm	Weight (gm)	8
Continuous current	20A		

As all the components were selected according to our requirements, the weights of all the components and overall weight were estimated, and it is given in Table 8.

**Table 8:** Component weight estimation.

Component	Quantity	Weight (grams)	Component	Quantity	Weight (grams)
Battery	1	136	FCB	1	30
Motor	2	67	Payload	1	100
ESC	2	16	Wires & Frame	1	140
Propeller	2	10	Total	500	

Finally, from the acquired data on the battery and the overall weight of the UAV, the Flight time was estimated to be 11.88 min and it is clearly illustrated in Table 9.

**Table 9:** Flight time estimation.

Specification	Chosen data	Specification	Chosen data
Battery capacity	1550 mAh	Overall Weight	460 g
Battery discharge	90%	Flight time	11.88 min
Battery voltage	11.1		

## Proposed methodology

### Computational model

**Figure 12:**
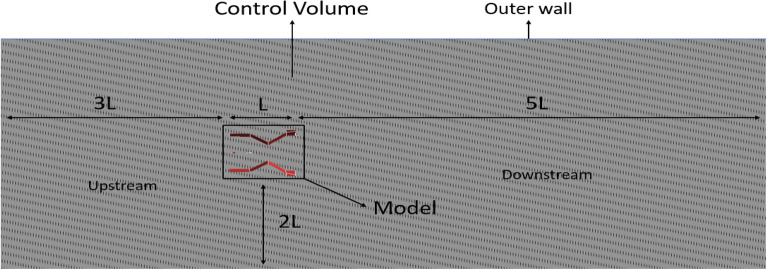
A systematic section view of the control volume used as computational model with the UAV.

The calculated design is constructed computationally, and it’s considered for further studies. The computational model comprises of two main designs i.e., the entire UAV and the control volume. Since our case deals with external flow, the control volume is constructed as there is need for an external volume which will be considered as the study area in case of aerodynamic flow analysis. In this scenario, the height of the UAV is the most significant dimension, hence it is chosen as the reference length for constructing the control volume. Regarding study, the appropriate size of the control volume can range from three to six times the reference length upstream, and from five to twelve times the reference length downstream. The control volume is chosen to have a cylindrical shape, although it might alternatively be rectangular. The outer boundary of the control volume will have greater relevance when it is situated at a considerable distance from the model. In such instances, the outer wall parameters will remain unaffected by the model’s response to input conditions. Therefore, the model is created for the purpose of conducting numerical analysis^30–40^. An FEA analysis is conducted on the computer model of the ducted drone. This method utilises the marketed model, in which material analysis is conducted by fluid structure interaction (FSI) and advanced computational study, taking into account various materials. The analysis is conducted using one-way coupling. In Fig. 12, the inner control volume spins while the outside control volume remains stationary.

### Discretization

**Figure 13:**
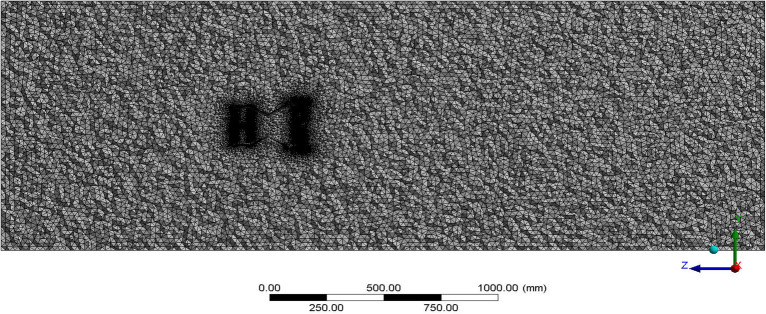
A typical section view of the discretized model of the UAV.

The complete model is numerically developed to facilitate the acquisition of an estimated result in a more efficient and expedient manner compared to human calculation. Therefore, the aforementioned model should be discretized in a manner that facilitates the solver’s ability to take it as input and solve it efficiently. The mesh can be categorized as either structured or unstructured, based on the level of complexity exhibited by the model. Structured meshes yield superior outcomes compared to unstructured meshes and also result in a reduced number of elements. However, in the majority of instances, the mesh will lack structure because to the characteristics of the model. The accuracy of the results approximation will be contingent upon the quality of the mesh. The use of an unstructured mesh can impact the computing time; however, employing a mesh that is concentrated around complicated regions is more suitable in comparison to a structured mesh. The mesh in close proximity to the model should have a high level of concentration in order to effectively analyse the fluctuations in boundary attributes of the condition. To do this, the model’s discretization can be finely adjusted using both global and local mesh settings. Figure 13 illustrates the domestically produced components following the discretization of the complete control volume. During the discretization process, it is important to evaluate the mesh attributes such as orthogonal quality, skewness ratio, and aspect ratio^30–40^.

### Boundary conditions

**Figure 14:**
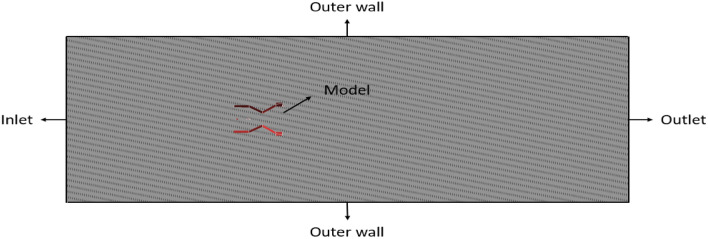
A systematic representation of the boundaries named for the computational model of the UAV.

Considering the application, the velocity was set at a constant value, so the intake is designated as a velocity inlet. The UAV will reach a maximum velocity of 20 m/s. There are three primary types of outlets: far-field, out-flow, and pressure outlet. The computational model can be shown in Fig. 14. In the far-field outlet, the characteristics are identical to those of the atmosphere. This condition is applicable when the model is positioned at a sufficient distance to prevent any effect from the model’s behaviour. When it comes to outflow, the solver automatically determines the properties at the outlet. However, with pressure-based outlet, the user has the ability to specify the desired pressure at the outlet. The outer walls can be considered to have free slip conditions, as they do not affect any changes in the outcome. The chosen model is a no-slip condition, where the solver treats it as an object within the control volume^30–40^.

The material selection is determined by its maximum pressure resistance, which is computed based on the highest velocity. A Finite Element Analysis was conducted with fixed supports and pressure was applied at specified points. The propeller is affixed to the hub and the entire propeller will be subjected to the pressure. The MRF technique utilizes RPM as the input condition for analysis and calculates the time-step depending on RPM. The outside control volume is stationary, whereas the inner control volume will undergo rotational motion around an axis relative to the model’s orientation^30–40^.

### Solver and governing equations

The equations that are used to solve the problem include continuity, momentum (in all directions) and Navier stokes equation. The solver type used here is Pressure based, since in our case we deal with incompressible flow of fluid. In this type of solver, the pressure inlet will give a higher convergence rate than that of the velocity inlet. In SIMPLEC scheme method, the pressure is assumed and solved for the velocity using momentum equations and then the continuity equation is checked. Based on the result the pressure gets modified and then it gets rechecked again by the solver. The type of coupling method imposed is coupled as it gives convergence with approximate result. The standard k-epsilon model with curvature correction was used to capture better at curvatures. The cell zone condition determines the operation environment properties like the type of fluid and its properties^30–40^.

### Grid independence study

**Figure 15:**
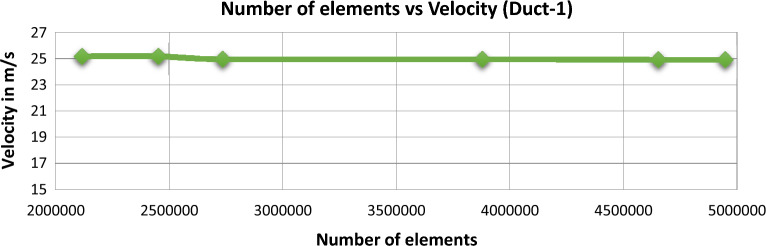
Comprehensive data on mesh independent study performed for Duct-1.

**Figure 16:**
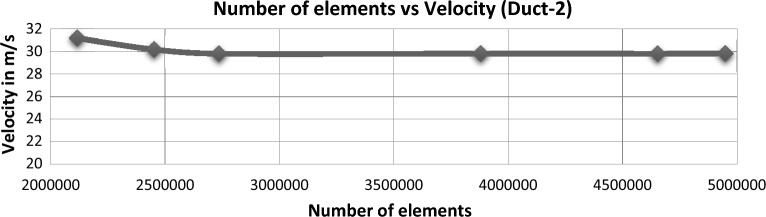
Comprehensive data on mesh independent study performed for Duct-2.

To obtain reliable outcomes for the CFD analysis of the UAV, a proper mesh need to be imposed on the computational model of the UAV. Henceforth separate grid independence study was conducted for both duct configurations of the UAV. Figures 15 and 16 represent the induced velocity on its respective element count for Duct 1 and Duct 2 respectively and then it is concluded that an element count of around 2,700,000 and above produced similar outcomes. So, the element count of 2,700,000 was chose to be the optimum mesh for the CFD analysis for both the cases.

### Validation studies-1

**Figure 17:**
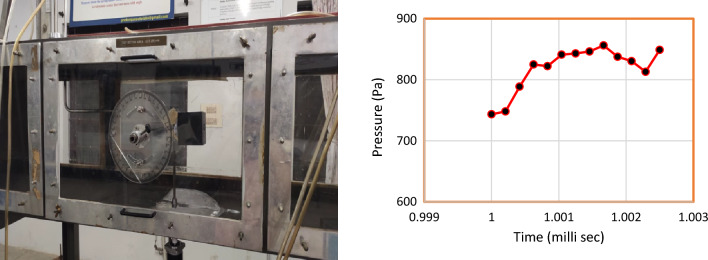
Typical view of the Duct placed in the test section of the wind tunnel and its graphical representation of pressure obtained from the wind tunnel testing during various time interval.

**Figure 18:**
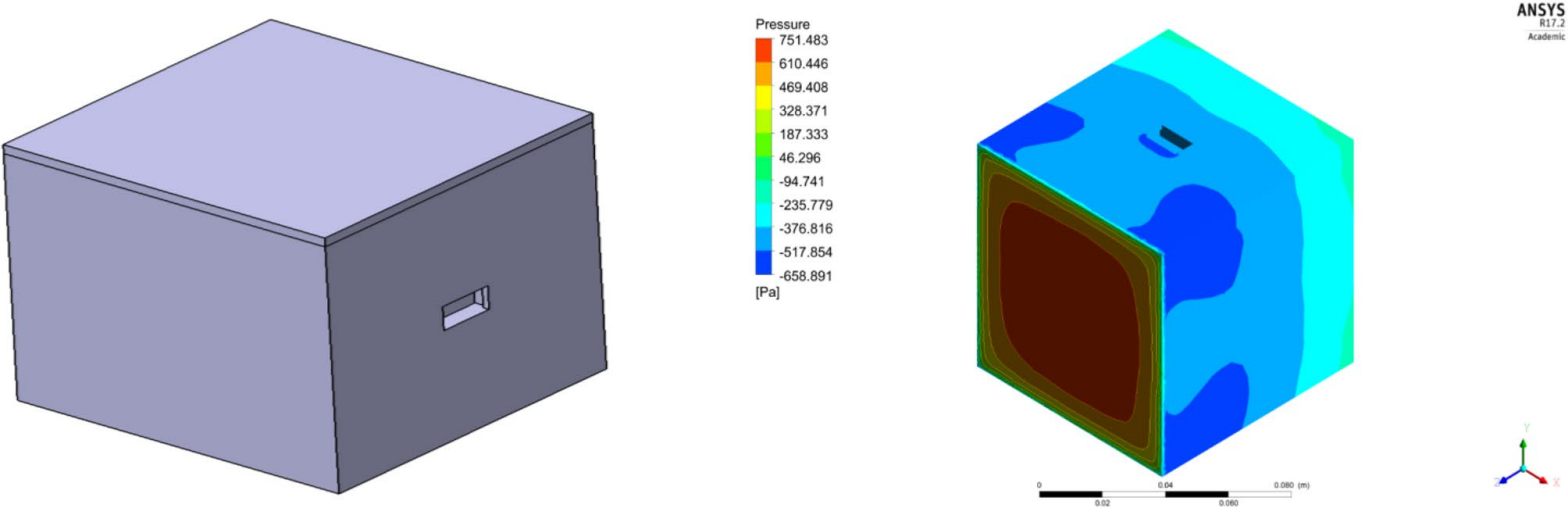
CAD model of the duct placed in a cube with pressure senor in its face and its computational outcome of the pressure acting on the duct.

For the aerodynamic analysis validation of the model, a C–D duct was placed inside a cube which has pressure sensors attached to it. The model is tested using a subsonic wind tunnel. This unique validation process was performed to specifically validate the dynamic pressure acting on the duct. A typical view of the model placed in the test section of the model and the graphical representation of the pressure outcomes obtained through the pressure sensors during the wind tunnel testing is represented in Fig. 17, and during the test the pressure sensor positioned on the cube’s face in the incoming flow direction recorded a dynamic pressure range between 740 and 775 Pa at the initial time period. On the other hand, the computational analysis provided an induced dynamic pressure of 751.48 Pa which can be observed from Fig. 18.

**Figure 19:**
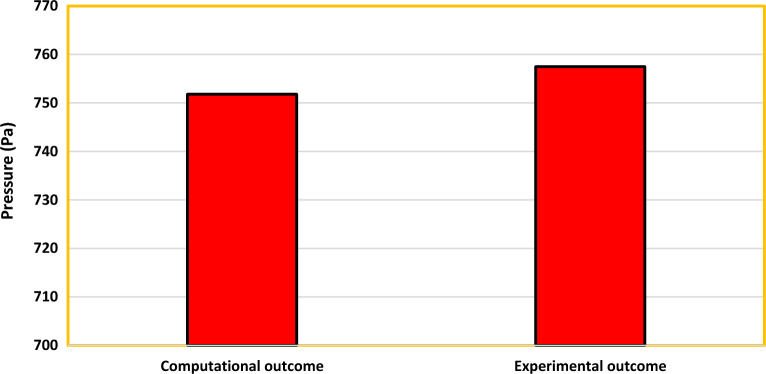
Comprehensive results obtained from computational and experimental studies.

From comparing both the outcomes depicted in Fig. 19, the error percentage was estimated to be around 0.8%. Thus, the imposed computational methodology is considered to provide appropriate reliable outcomes. The validation for the MRF imposed for the propellers which are validated in^40^ were inferred.

### Validation studies-2

**Figure 20:**
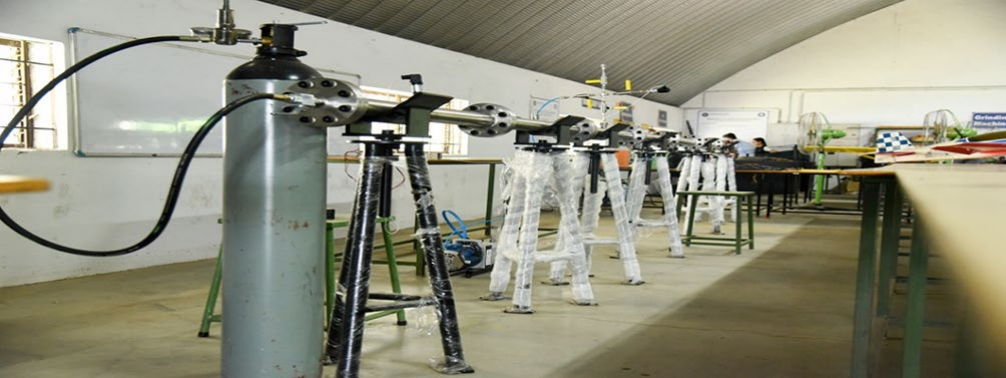
Test setup used for high pressure load development.

**Figure 21:**
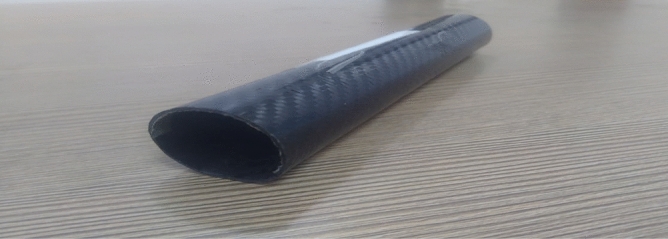
A typical view of test specimen before structural failure made of CFRP composite.

Validation on the collected structural computational outputs through experimental testing is required in order to evaluate the accuracy of the enforced advanced structural simulations utilizing ANSYS Workbench 17.2. In this study, the authors compare the results of tests carried out at a facility with a high-velocity jet to the results of computational structural procedures that were required to be carried out. Figure 20 provides a visual representation of a facility including a high-velocity jet. Figure 21 shows the imposed test specimen made of carbon fiber reinforced polymer in the ducted shape.

**Figure 22:**
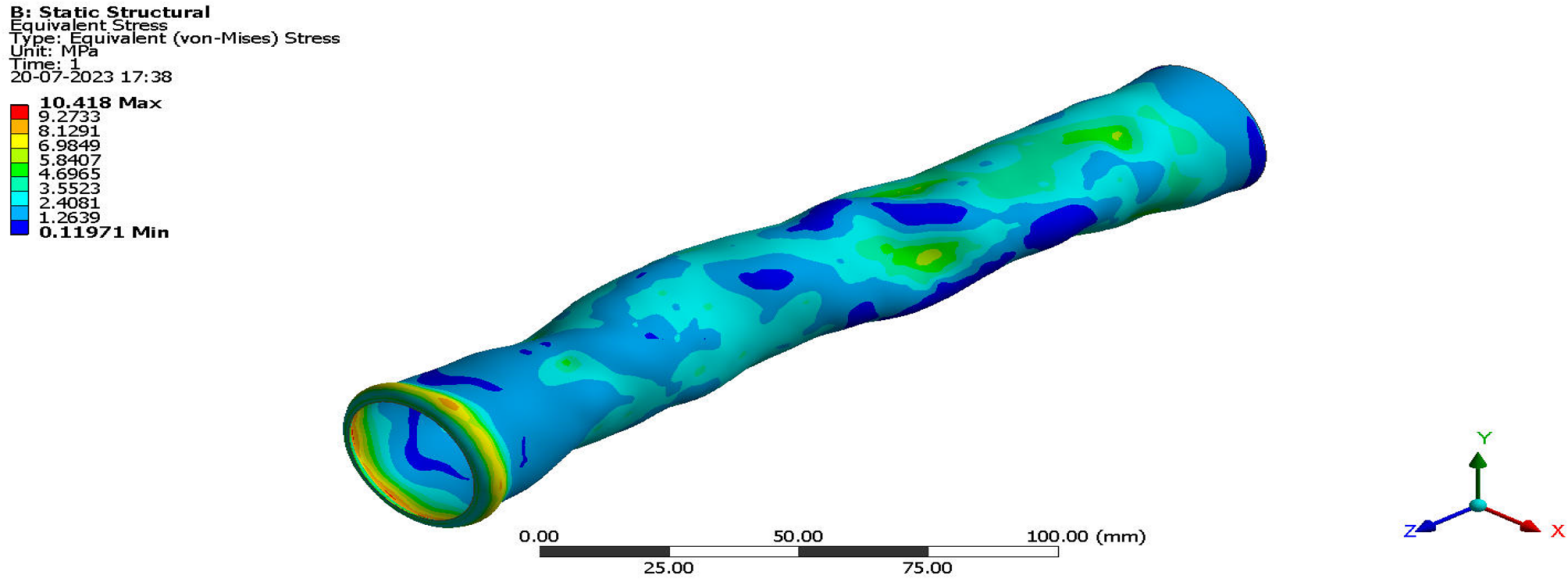
A typical isometric view of equivalent stress acting on CFRP-WN-PRP.-230 based test specimen.

**Figure 23:**
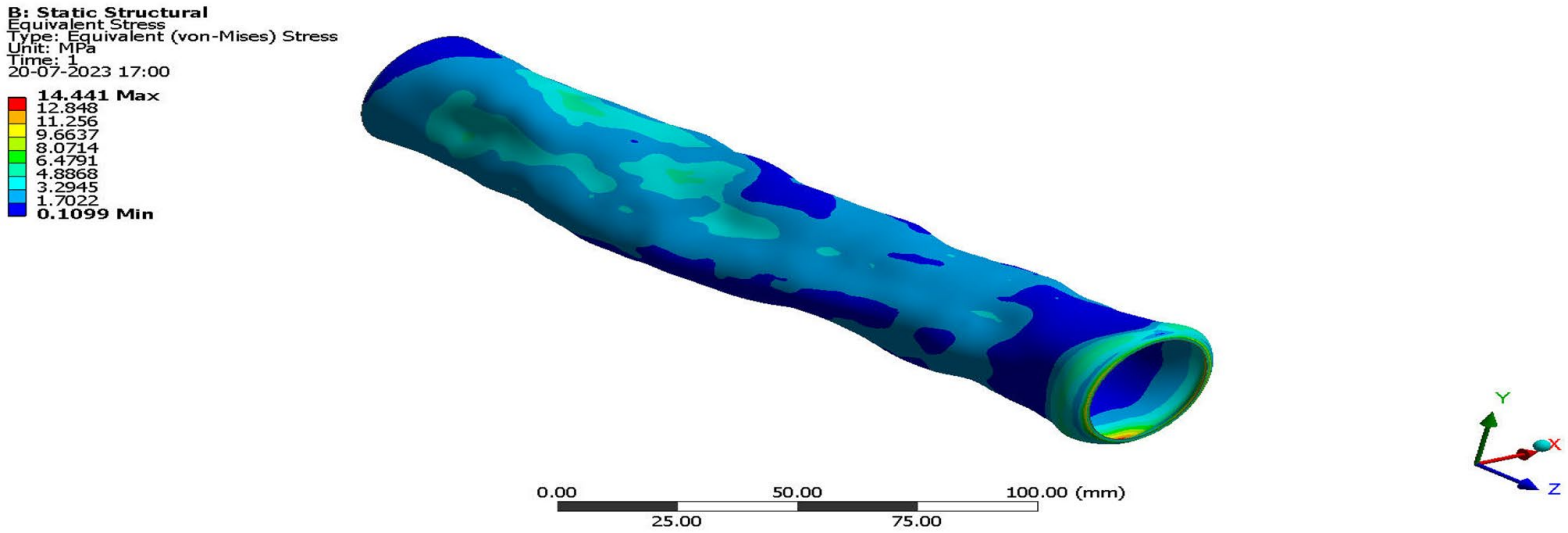
A typical isometric view of Equivalent Stress acting on CFRP-UD-PRP.-230 based test specimen.

The experiments are conducted in an environment that accurately reflects the conditions outside the jet’s path, but the pressure inside the jet’s path is considerably greater. The test specimen made of carbon fibre reinforced polymers (CFRP) fails under the intense aerodynamic load of the 40-bar high pressure supply. However, the CFRP test specimen remains structurally intact throughout the experiment. Previous experimental results established that the woven CFRP had an ultimate stress of 3500 MPa. Once the experimental tests have been successfully conducted, the subsequent phase involves utilizing ANSYS Workbench 17.2 to do the computations required for the tests to be deemed genuine. A newly developed computational platform, which incorporates a high-speed jet route and computational test specimen, is capable of generating precise representations of experimental test setups and computational test specimen design data. This is achieved by connecting the high-velocity jet pathway with the computational test sample. The computer simulation included pressure inlets and a discretized model to determine the necessary pressure fluctuation on the test specimen. ANSYS Workbench 17.2 employs a sophisticated system coupling approach to transfer the expected aerodynamic pressure from the model to the computationally generated models of aluminium alloy and CFRP-based composites. Furthermore, the FEA-based solver is employed to calculate the maximum equivalent stresses for both models under applied and transmitted aerodynamic loads. Figures 22 and 23 depict models constructed from CFRP and their corresponding structural consequences, respectively, to provide a clearer explanation of the structural repercussions.

Using this computational method, a maximum equivalent stress of 10.23 MPa is calculated for the woven CFRP, while a maximum equivalent stress of 14.441 MPa is calculated for the improved CFRP-UD-Prepreg based composite. Through the use of the imposed advanced FSI method, the authors found that both the CFRP computational test specimens exhibit acceptable stresses only and so the ducted structure would not break. In the experimental test setup too, both the specimens made of CFRP are unbroken. Both test specimens were made of test materials. Therefore, the decision to impose certain computational techniques on the various UAV components has been made, and one can rely on them to give accurate results. This is due to the fact that the decision has been finalized.

## Result and discussions

Starting with the determination of design parameters and component selection, followed by the complete design of the UAV using CATIA, all these steps contribute to the examination of the aerodynamic performance of both UAV designs. Following the investigation, both designs are evaluated and compared to determine the superior performer.

### Comparison of duct configurations

**Figure 24:**
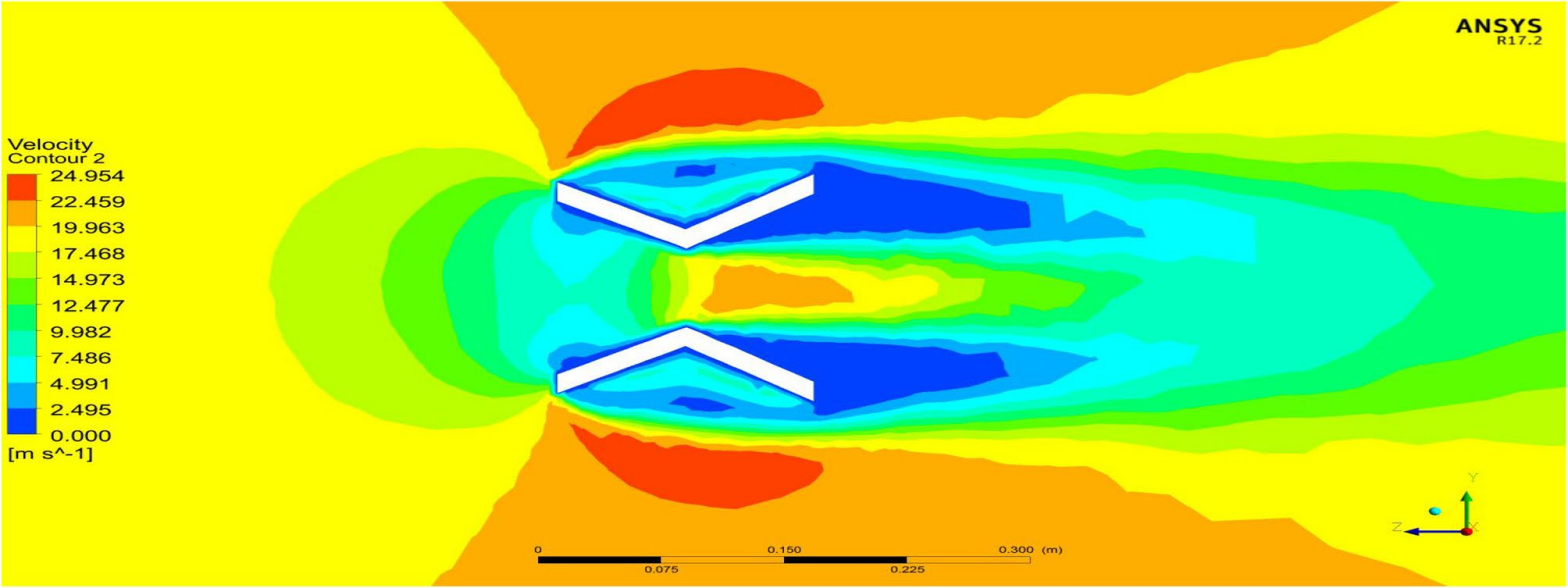
A typical planar view of velocity variation on Duct 1.

**Figure 25:**
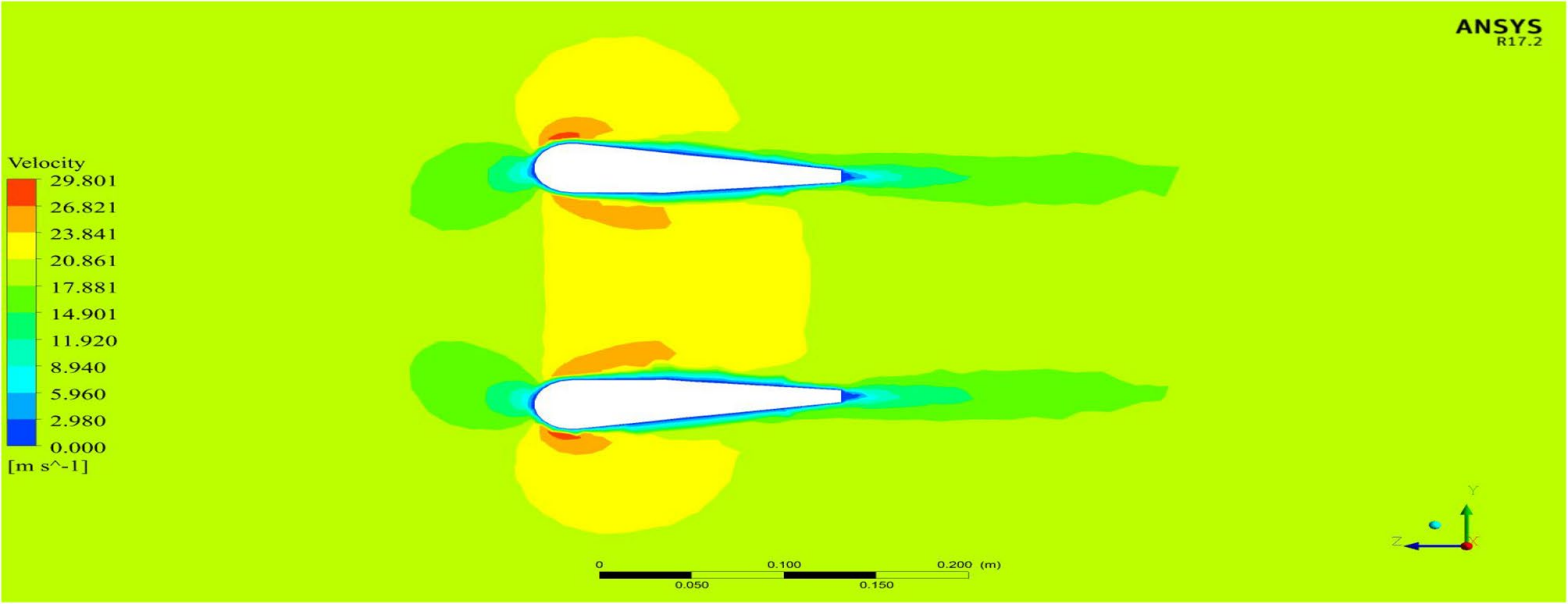
A typical planar view of velocity variation on Duct 2.

**Figure 26:**
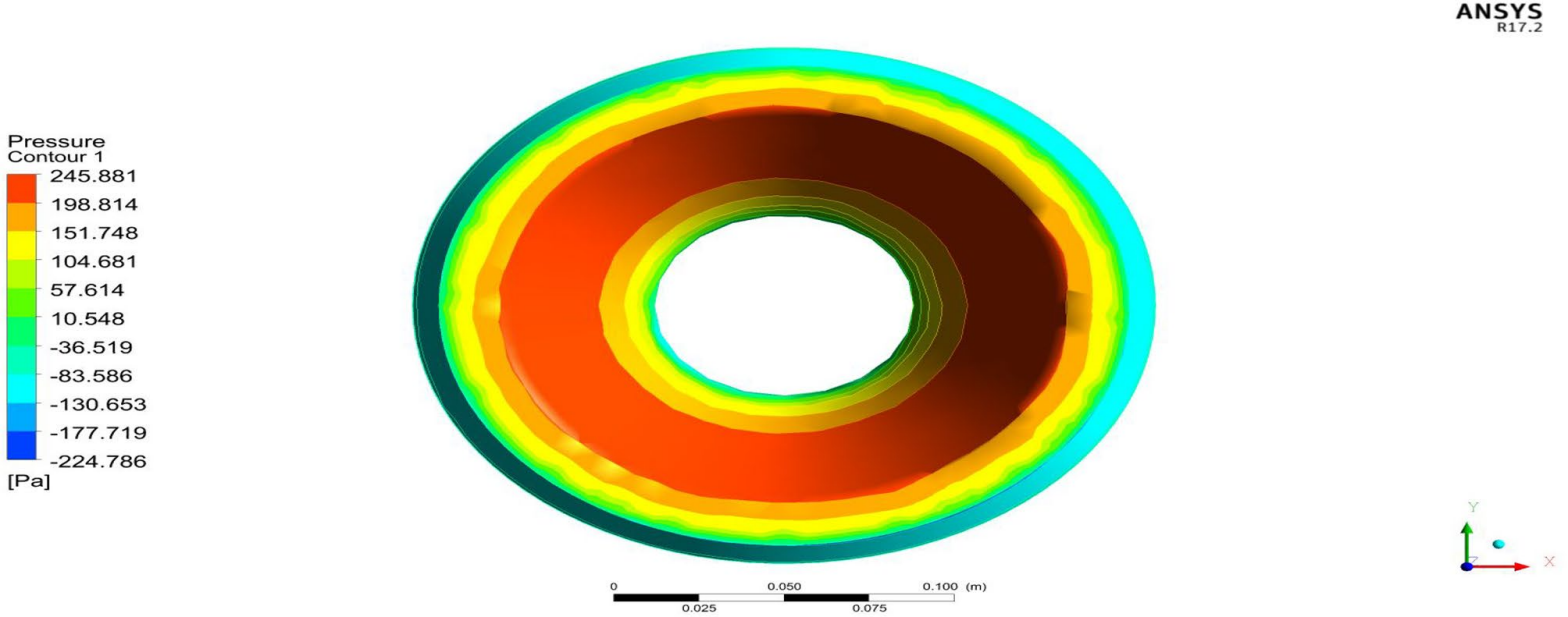
A typical top view of pressure distribution on Duct 1.

**Figure 27:**
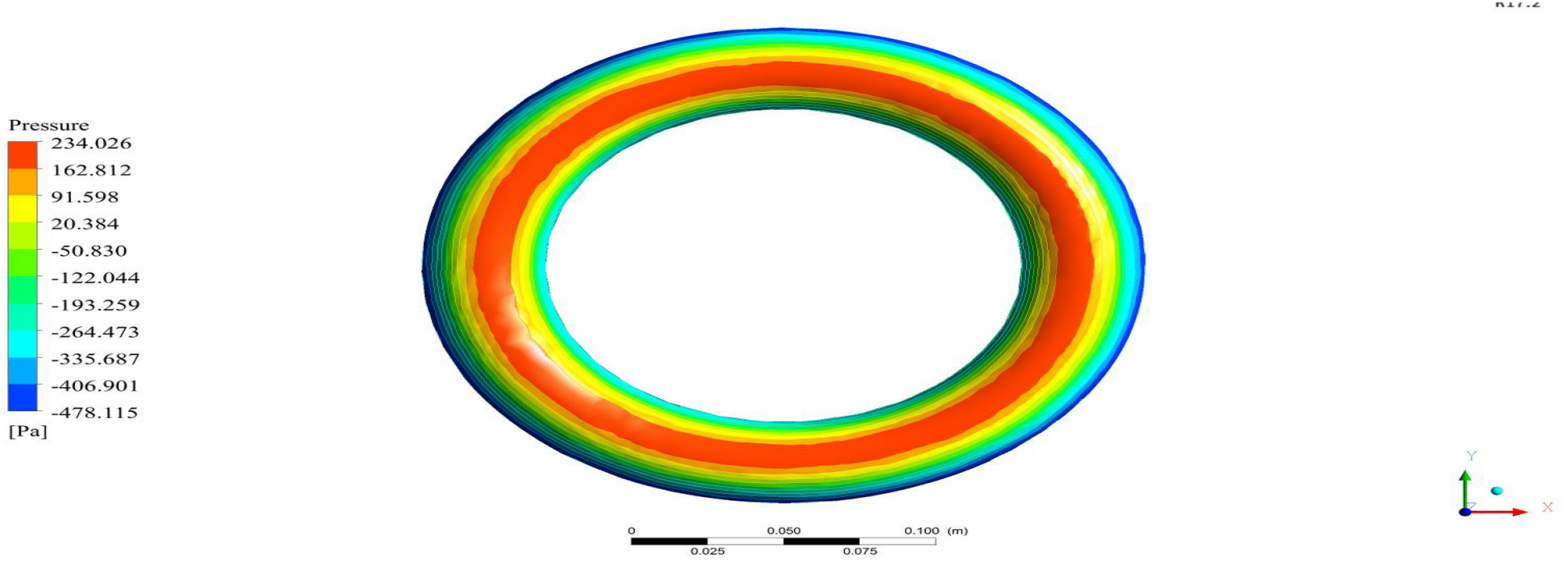
A typical top view of pressure distribution on Duct 2.

Starting with the ducts, both the ducts were subjected to aerodynamic load under appropriate boundary conditions and analyzed using Ansys 17.2. The resulting contours of the analysis are displayed in Figs. 24, 25, 26 and 27. These figures depict the pressure and velocity variations inside, on and over both the duct designs^30–40^.

Based on the results, Duct 1 increased the inlet velocity to 22.5 m/s and Duct 2 increased the inlet velocity to 26.8 m/s which is around 19% higher than Duct 1.Whilecomparing the pressure variations, 245.8 Pa pressure was observed at the converging section of the Duct 1 and 234 Pa of pressure is observed at the top of the Duct 2 and it is around 4% lesser than Duct 1.This clarifies that duct 2 performs better than the other by increasing the velocity and allowing less pressure to act on its structure. This duct contributes to the overall thrust production of the drone along with its coaxial propulsive system. This helps the drone to climb higher altitudes quicker.

### Comparison of deflectors based on angle of attack

**Figure 28:**
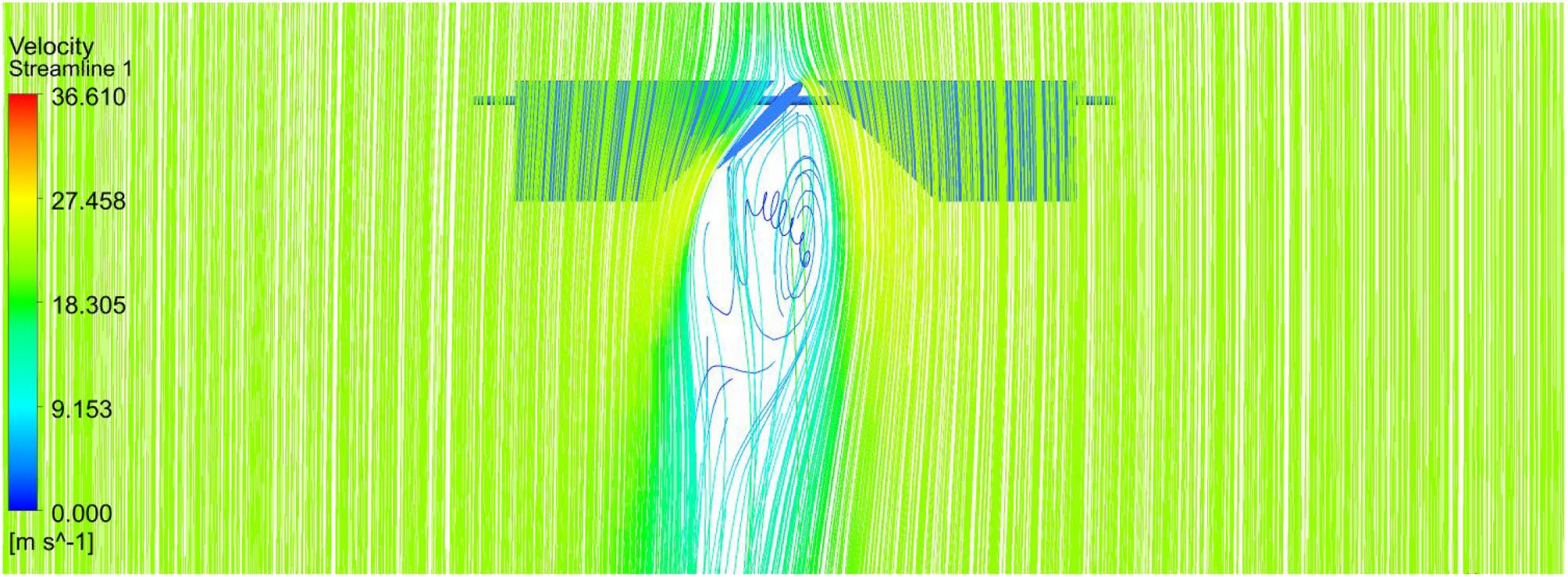
A systematic top view of velocity variations due to deflector at 45 degrees of Duct 1.

**Figure 29:**
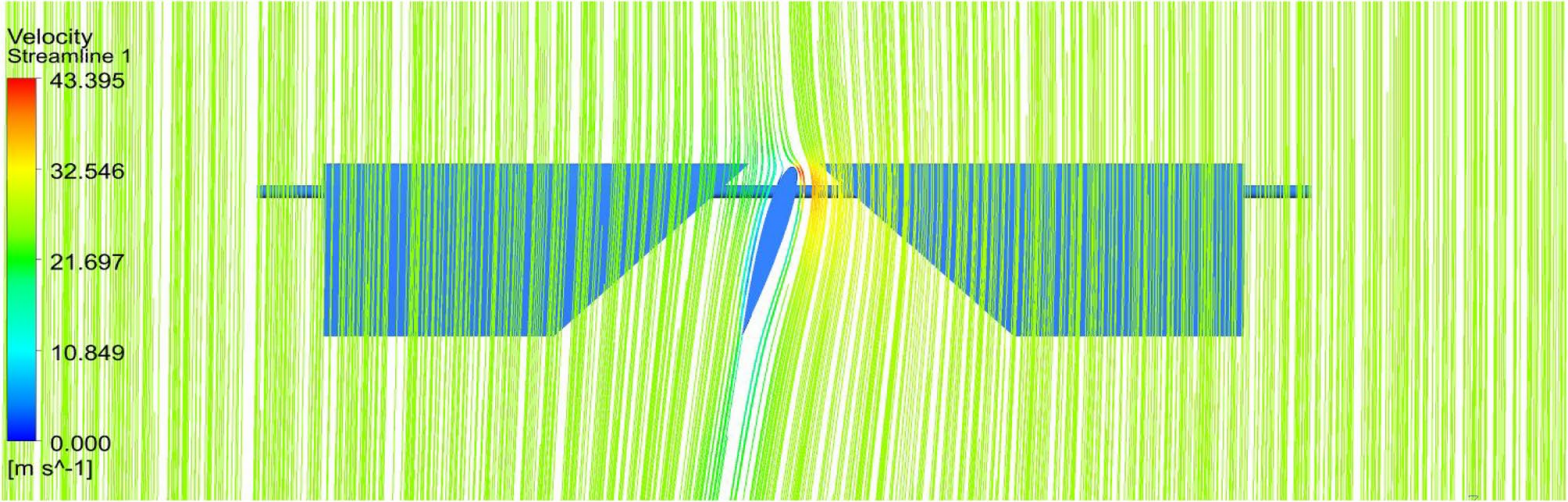
A typical top view of velocity variations due to deflector at 15 degrees of Duct 2.

The deflectors are a significant component because they act as the control surface for our UAV. They are used to maneuver the UAV by deflecting the flow of air passing out of the duct. Because of their importance they were separately analyzed to evaluate their effectiveness. From the results in Figs. 28 and 29, it is clearly visible that the aerodynamic load acting on the deflectors can create pressure and velocity variations on and between their surfaces which will generate required lift and side forces^30–40^.

Based on the outcome of the steady flow analysis the amount of Lift, Drag and side forces are compared. From Tables 10, the amount of Lift force that is required by the deflector to rotate the entire UAV to attain maneuvering was attained at a minimum of 15 Degrees. The maximum amount of lift required is attained before the aero foil profile reaches its stall angle, so that the drag at maximum angle of attack is avoided. The other higher angle of attacks was considered initially for Lift attainment but later it will be used in some cases where we require sudden drag at certain operating conditions as they produce more Drag compared to lift^30–40^.

**Table 10:** Forces on deflectors—Duct 1 and Duct 2.

	Duct 1		Duct 2	
AOA	Lift (grams)	Drag (grams)	Lift (grams)	Drag (grams)
15	112.8520	− 33.2835	162.002	− 46.8152
30	100.3911	− 7.1992	143.739	− 102.0226
45	95.6188	− 116.91	136.755	− 166.4279

From Table 10 it can be inferred that Duct 2 deflectorsproduce around 30% more lift than Duct 1 deflectors in all three different cases of varying angle of attacks. From both the duct analysis and the deflector plate analysis it is clear that Duct 2 performs better than Duct 1 in all the imposed conditions.

**Figure 30:**
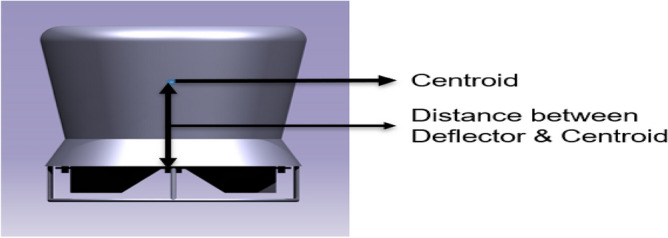
A schematic representation of centroid and location of deflector.

**Figure 31:**
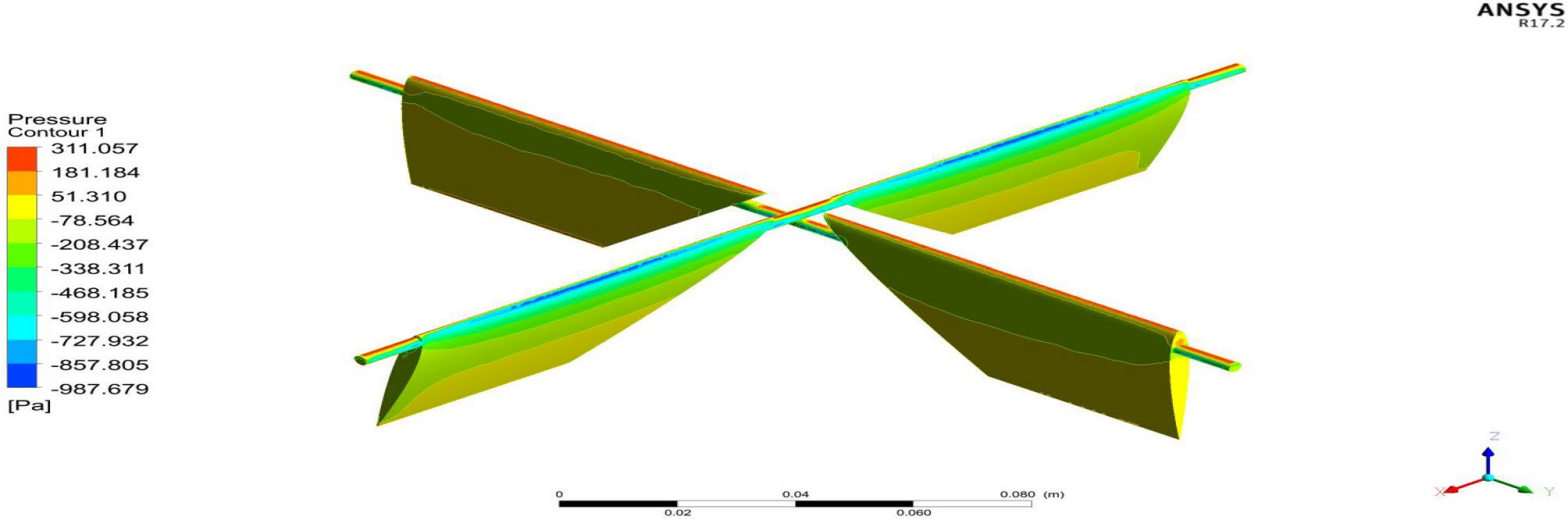
A typical isometric view of pressure distributions on the deflectors of Duct 1 at 15 degrees.

**Figure 32:**
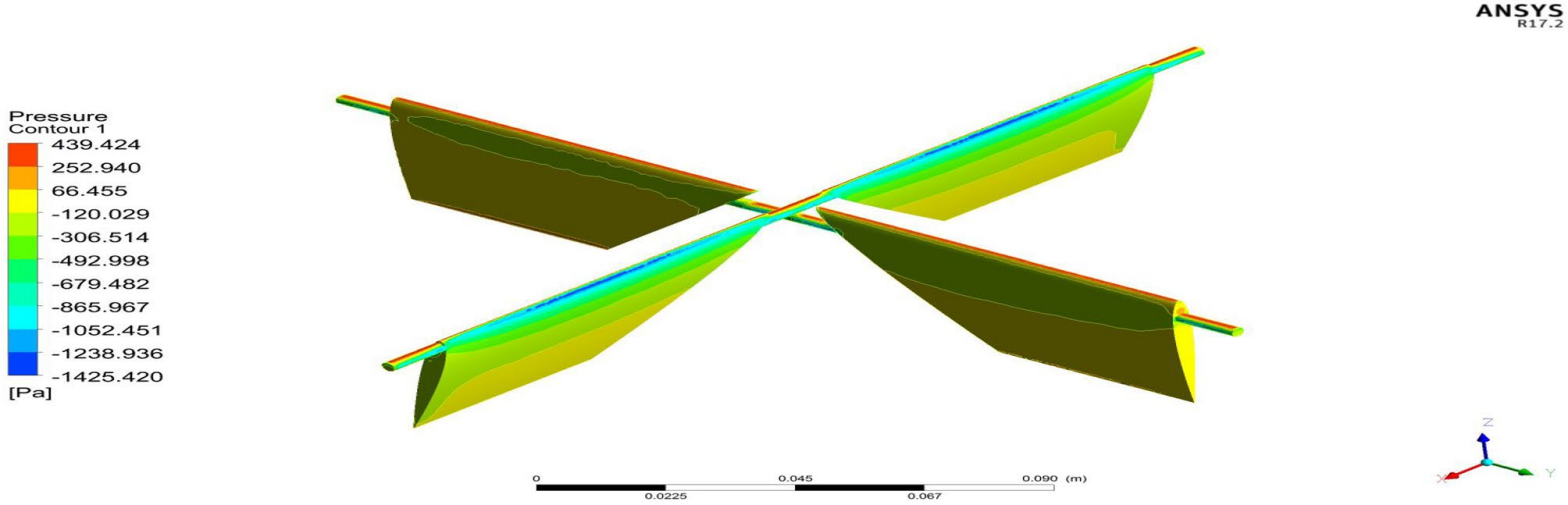
A typical isometric view of pressure distribution on the deflectors of Duct 2 at 15 degrees.

Based on Table 11, the moment created by the deflectorvaries depending on another factor i.e., the distance between the deflector plate and the centroid of the entire object which is displayed in Fig. 30. With the product of the force (lift) and the centroid distance, the moment is calculated and is depicted in Table 11 to attain the force required to achieve our directional changes i.e., rolling and yawing by moving the plates to certain angle of attacks to achieve those fore said manuevers. From Table 11 it is clear that the deflector plates attached to Duct 2 creates higher moments which is very useful for the control of the UAV as it can provide higher effectiveness with a minimum work possible from the actuators. The aerodynamic pressure impacts on the deflector plates with different orientations are systematically revealed in Figs. 31 and 32^30–40^.

**Table 11:** Comprehensive study of moment created by the drone with respect toits centroid.

AOA	Duct 1 Moment (Nm)	Duct 2 Moment (Nm)
15	132.60	197.34
30	117.95	175.09
45	112.35	166.58

### Vertical Take-off

**Figure 33:**
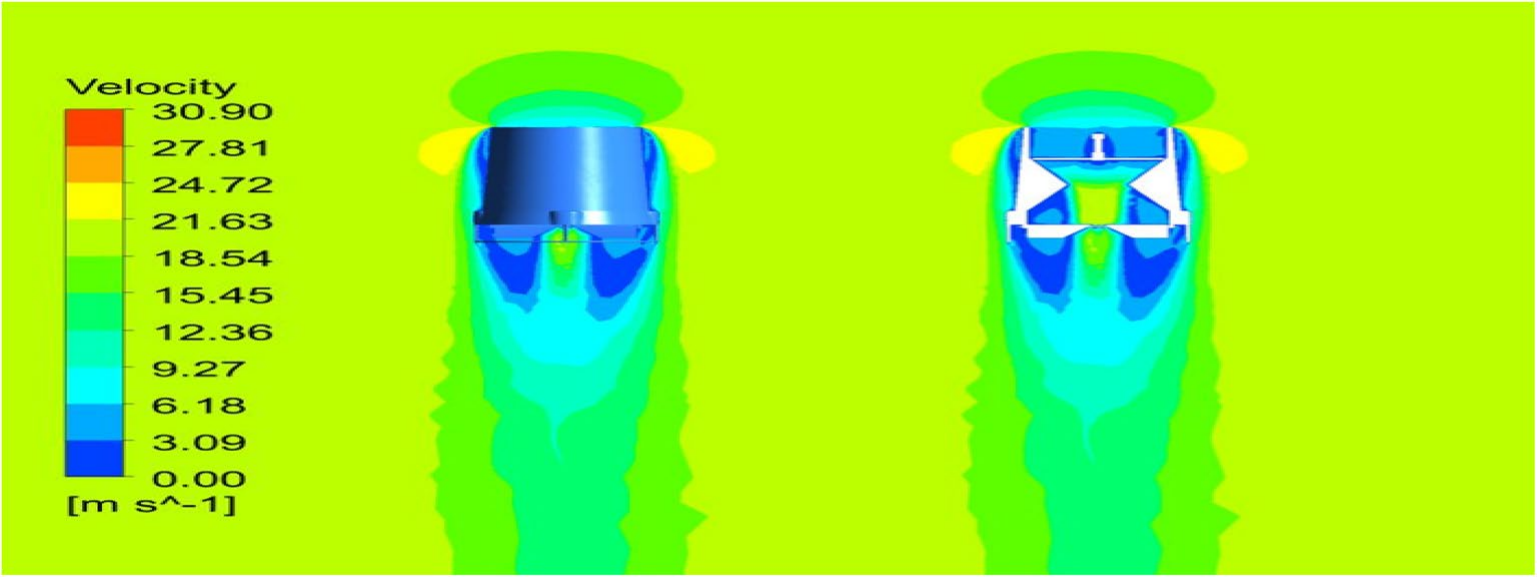
A typical planar view ofvelocity variations over Design 1.

**Figure 34:**
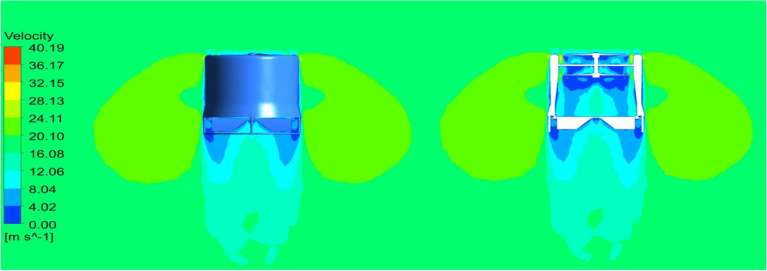
A typical planar view of velocity variations over Design 2.

Most part of the time the UAV will be in VTOL position and hence, a steady flow over the entire UAV was analyzed and the results were obtained as in Figs. 33 and 34 and the pressure and velocity variations on both the UAV designs displayed in the figures were observed. From the Figures it can be noted that the ducts help the drone to increase the velocity at the exit area, which helps the drone to take-off quickly as mentioned in the previous sub section^30–40^.

### Deflector angle − 45 Degree

**Figure 35:**
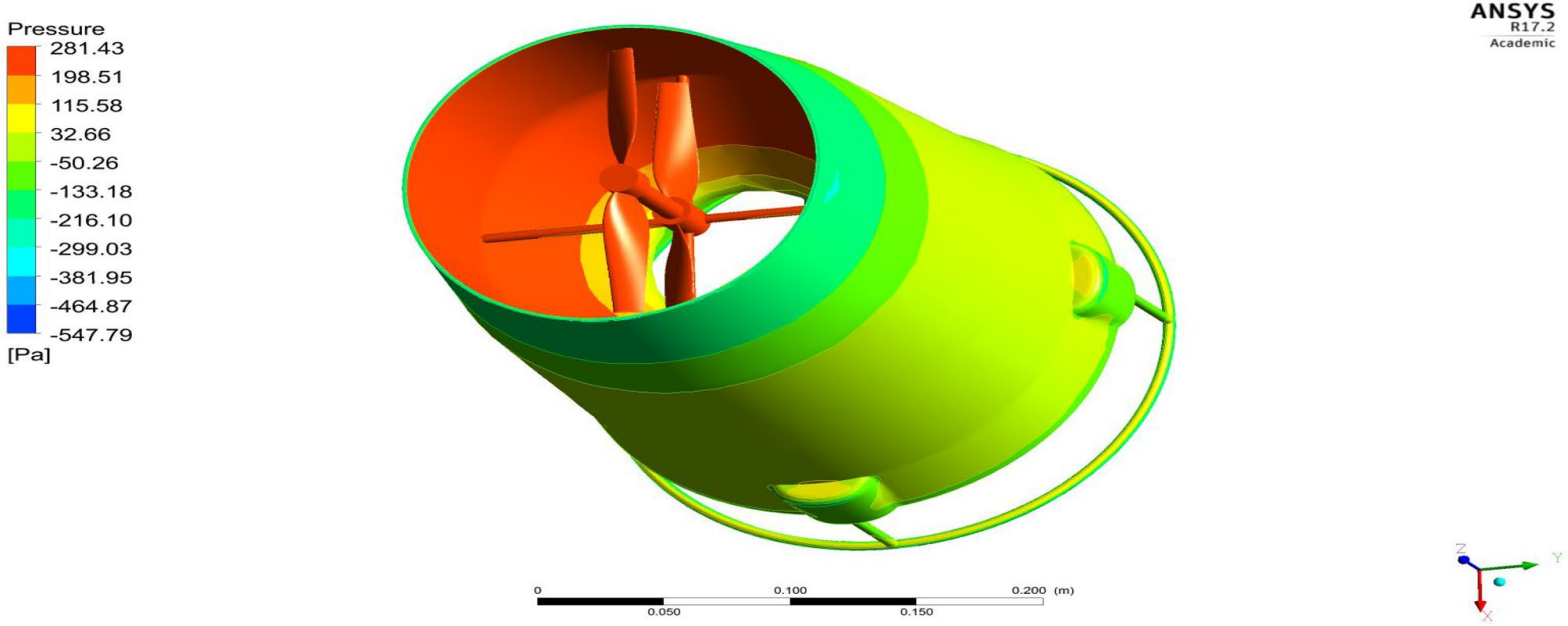
A typical isometric view of pressure distribution on Design 1.

**Figure 36:**
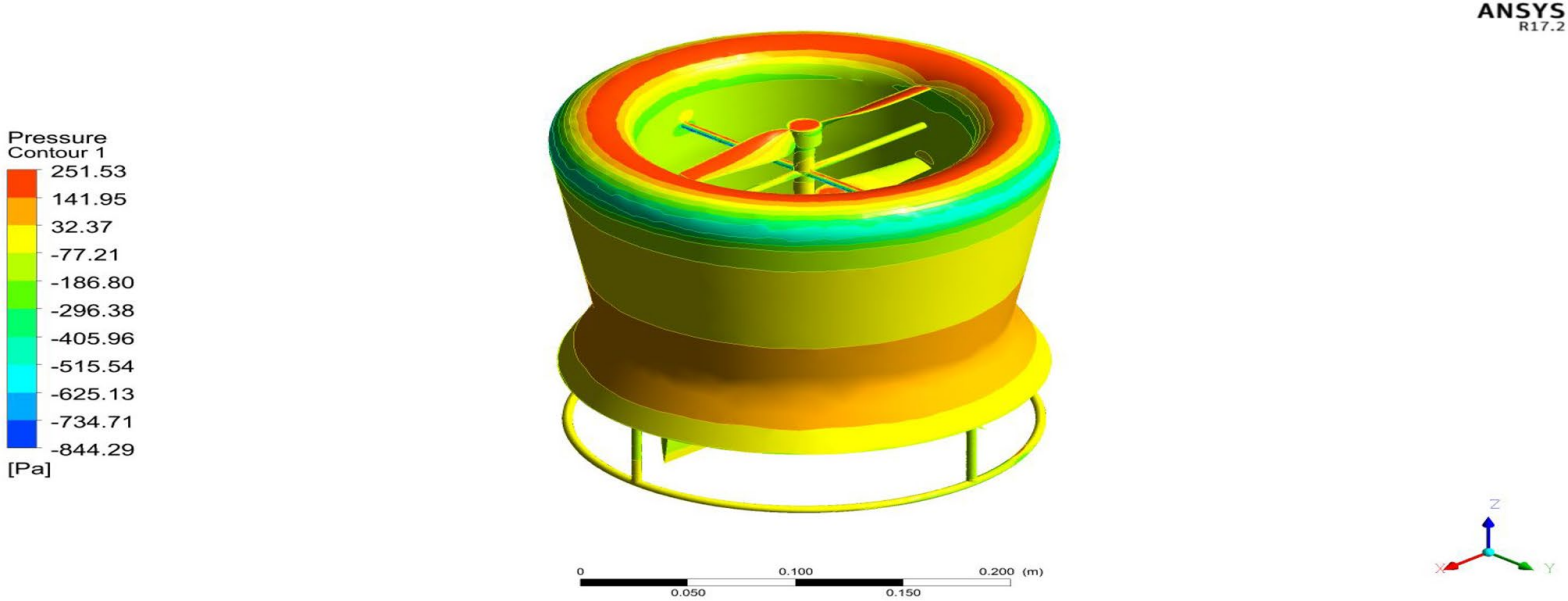
A systematic isometric view of pressure distribution on Design 2.

The maximum deflection angle of the deflector plate is 45 degrees and hence a steady case analysis was performed on the model having the deflector being deflected for about 45 degrees. The results of the analysis are shown in Figs. 35 and 36, in which the velocity variations over the UAVs and the pressure distribution on the UAVs are clearly observable and recorded for further optimizations^30–40^.

### Cruising condition

The secondary phase of mission profile of the UAV is to cruise. To do so the moment created by the deflecting plates plays the main role. The maximum deflection of the UAV is assumed to be 45 Degree which would be the maximum requirement to achieve directional changes. Hence, a static analysis was performed on the model to read the velocity and pressure changes^30–40^.

**Figure 37:**
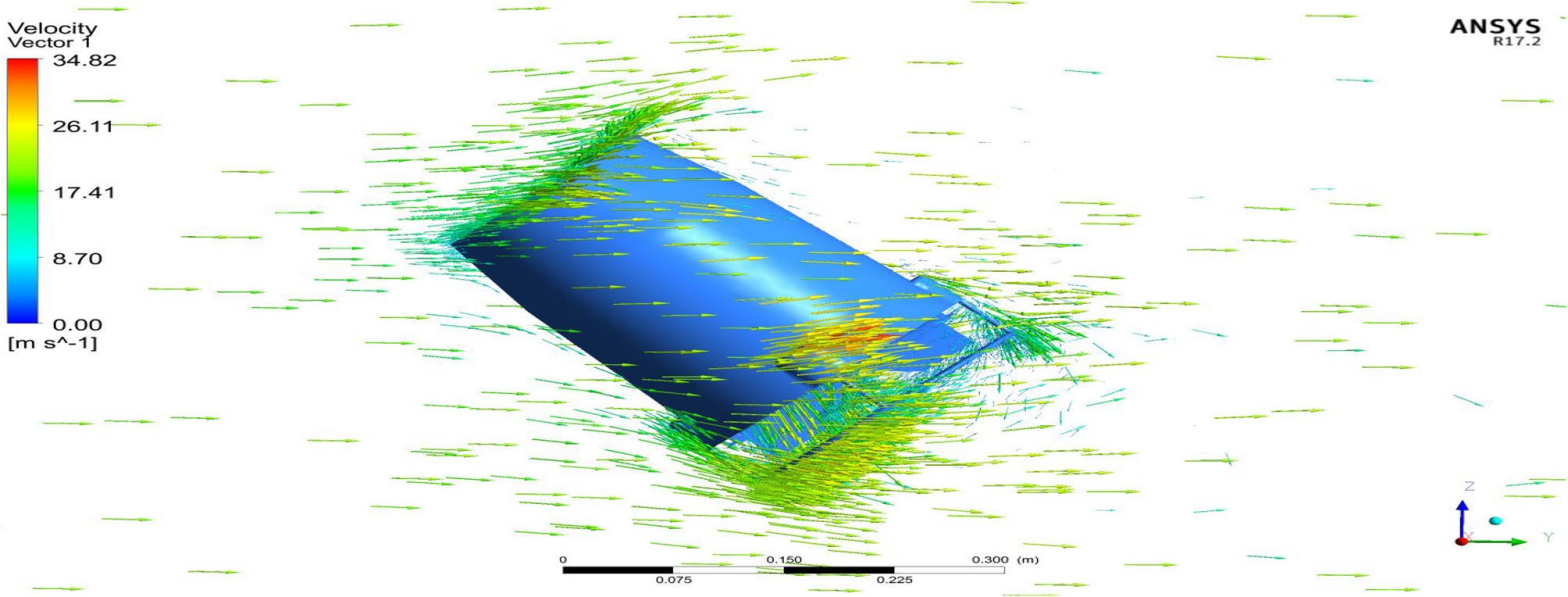
An emblematic view of velocity variation overDesign 1 of the UAV.

**Figure 38:**
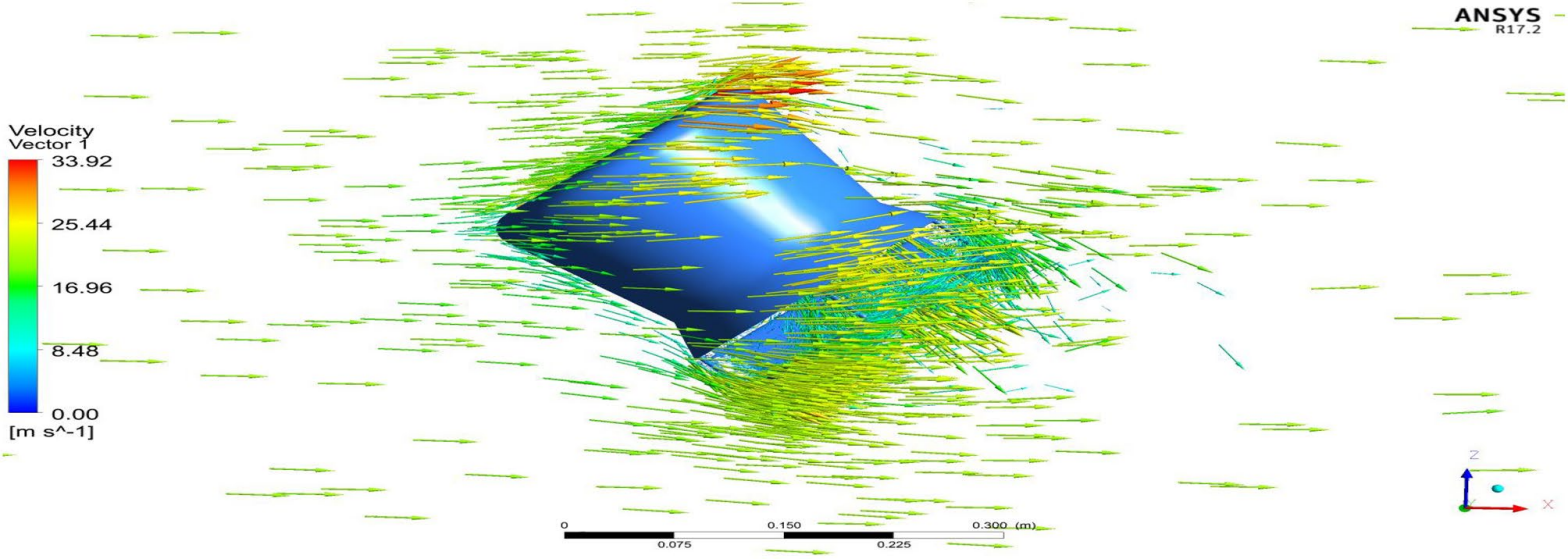
An emblematic isometric view of velocity variations over Design 2 of the UAV.

As a result, we can observe the region where there is maximum pressure and velocity generated during the movement in Figs. 37 and 38, which is noted for design optimization. The cruise condition can be performed with complete stability and the drone can stabilize itself even if there are any occurrences of external disturbances. This ability of the drone is analyzed and verified in the control dynamics section of this research.

### Moving Reference frame

The Moving reference frame technique is more popularly used for simulating problems which involve rotational motion. In this approach a simple actuator disc model is prepared and inside that model, in our case the propeller is arranged inside the disc. So ultimately when the disk spins with an RPM, numerically it will be considered as the flow is rotating in that particular domain, which eventually brings the rotor effects to the flow. Our main focus is the propeller as the design is entirely based on it^30–40^.

**Figure 39:**
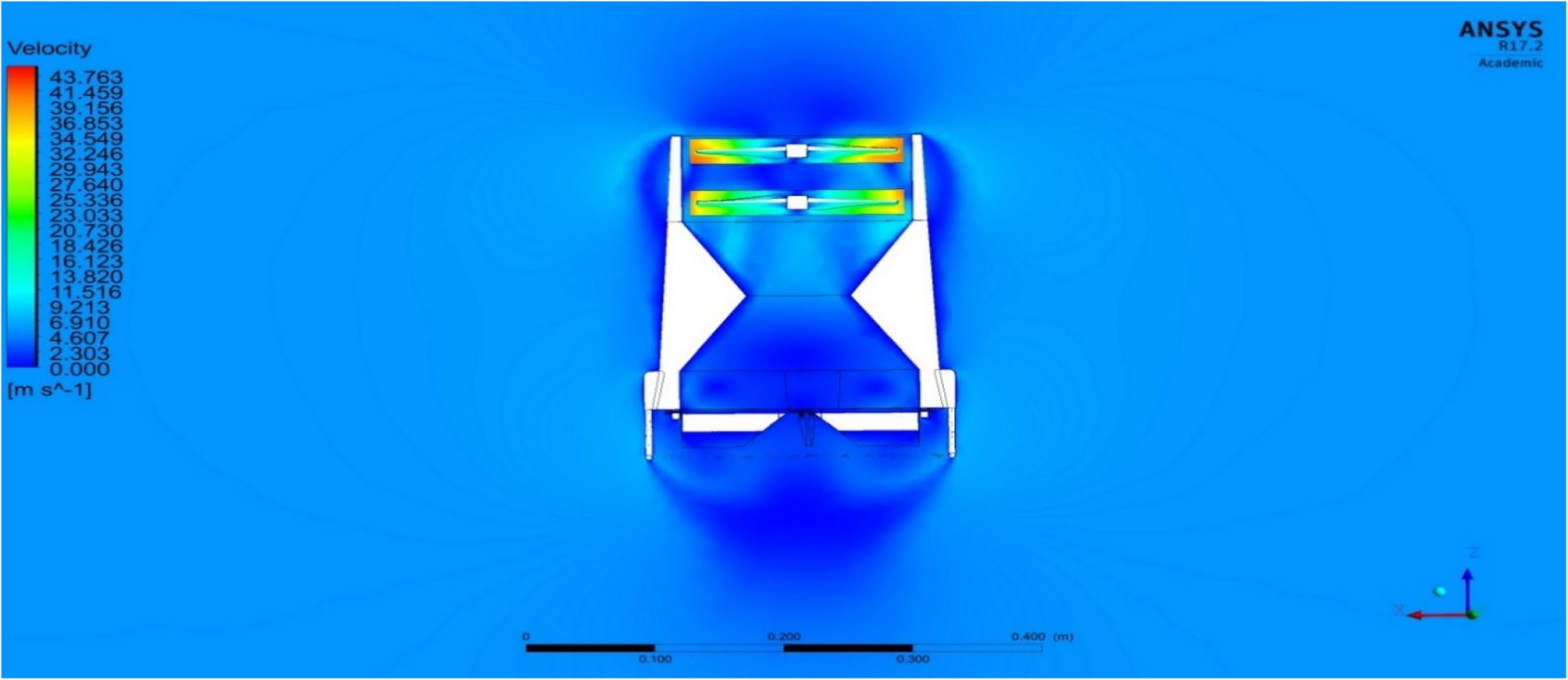
A typical planar view of velocity variationon the UAV.

**Figure 40:**
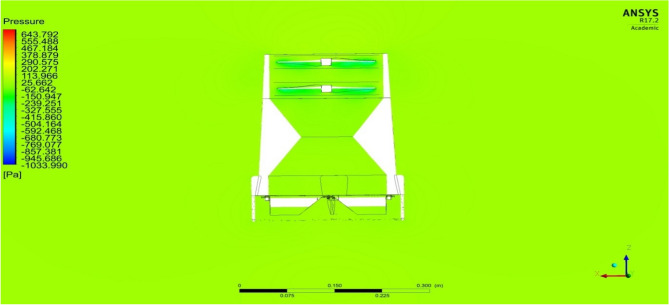
A systematic planar view of pressure distributions on the UAV.

The outcome of the above analysis is mainly focused on the effect of the upper propeller on the lower propeller because our case deals with the co-axial propulsion system and the combined effect of this system on the UAV. The velocity at the tip of the propeller is the main focus so as to reduce the tip loss. This case shows that there is a reduction in velocity when the duct is placed around it as it will not allow the tip flows to move further outward. The combined effect of the propellers can be observed in the pressure difference and velocity variation as represented by Figs. 39 and 40.

### Fluid Structure interaction

**Figure 41:**
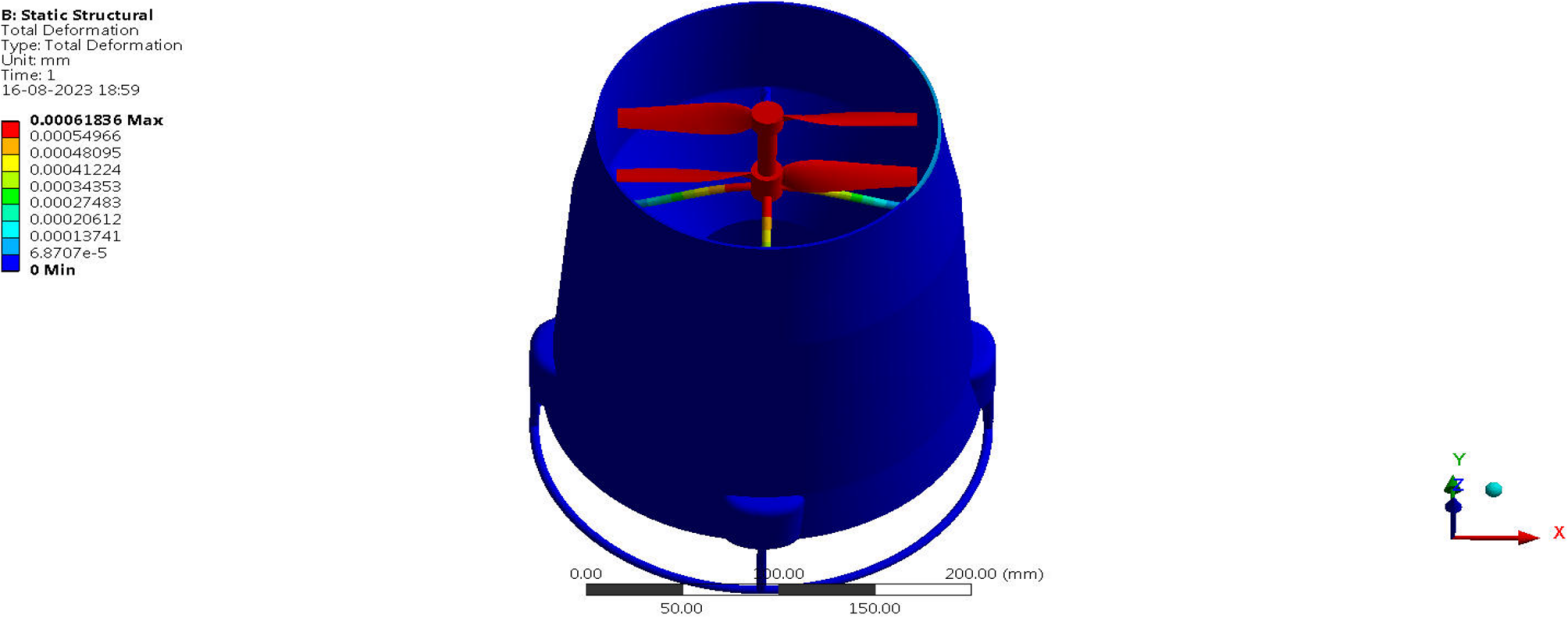
A typical top view of deformed structure of the UAV of FR-4 based composite.

**Figure 42:**
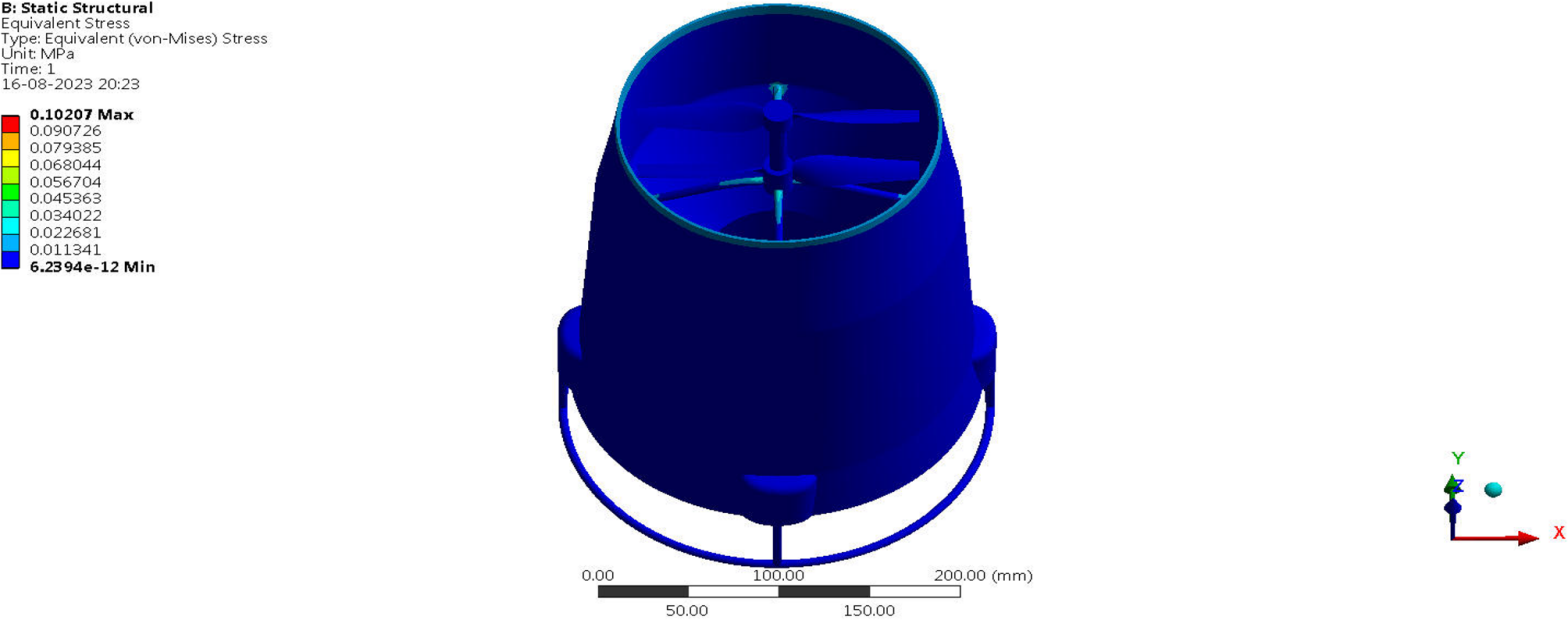
A systematic top view of equivalent stress acting on the UAV of Titanium alloy.

**Figure 43:**
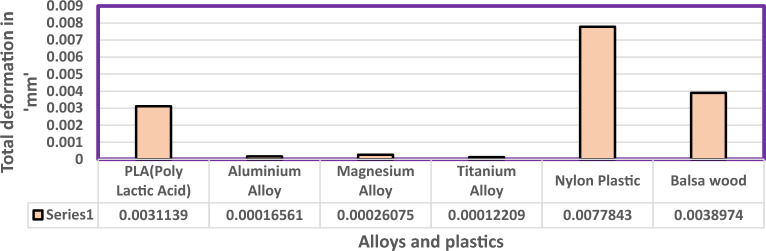
Comparative analysis of deformation for Alloys and plastics.

**Figure 44:**
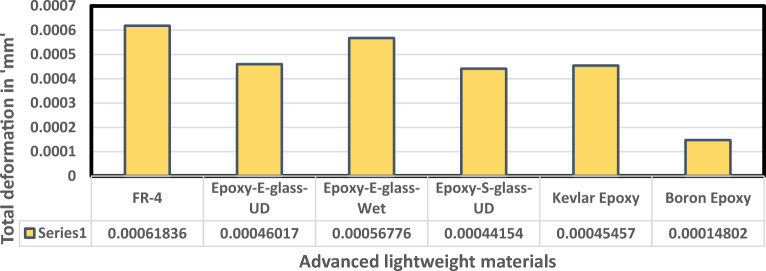
Comparative analysis of deformation for various advanced materials.

**Figure 45:**
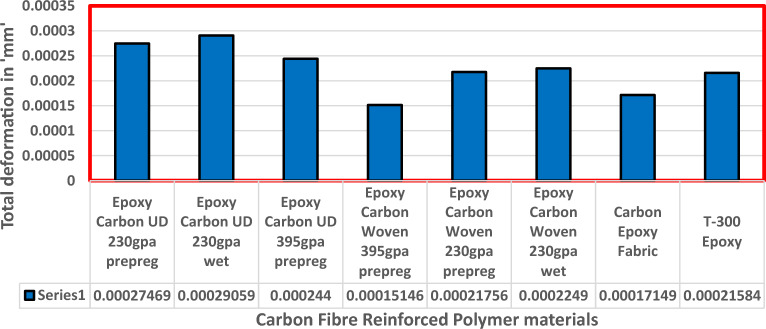
Comparative analysis of deformation for Carbon Fibre Reinforced Polymer materials.

**Figure 46:**
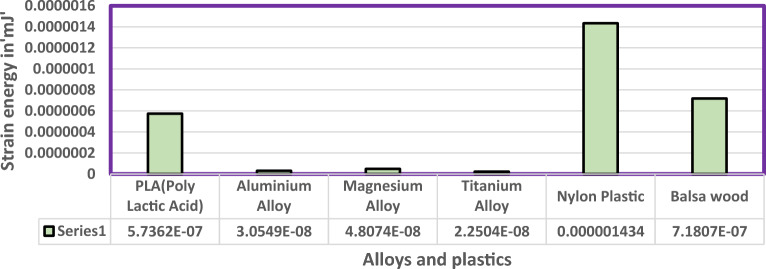
Comprehensive analysis of Strain Energy for alloys and plastics.

**Figure 47:**
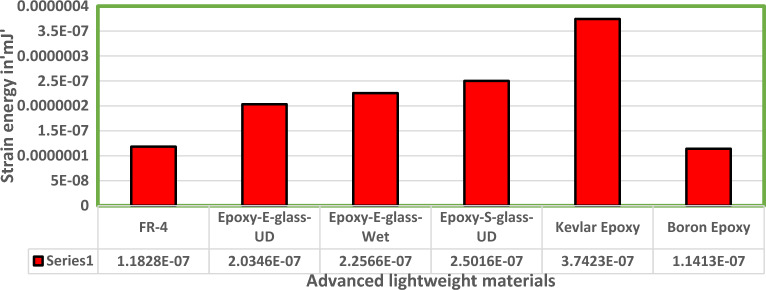
Comprehensive analysis of Strain Energy for lightweight materials.

**Figure 48:**
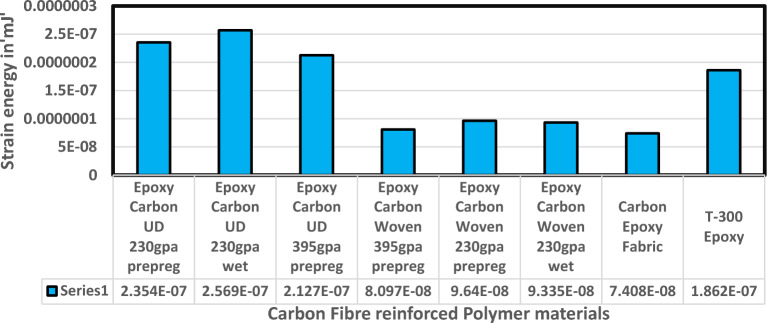
Comprehensive analysis of Strain Energy for carbon reinforced polymer.

**Figure 49:**
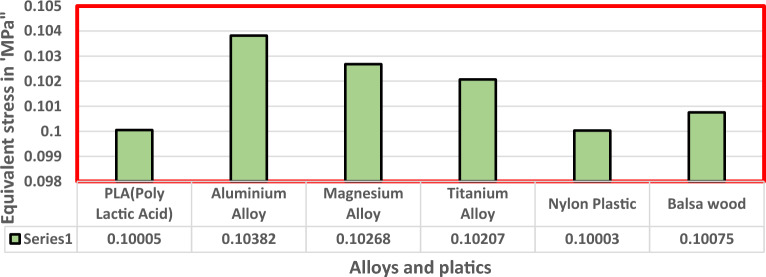
Comprehensive analysis of Equivalent stress on alloys and plastics.

**Figure 50:**
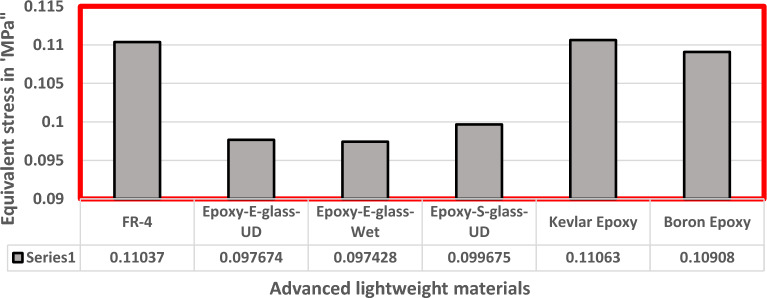
Comprehensive analysis of Equivalent stress on lightweight materials.

**Figure 51:**
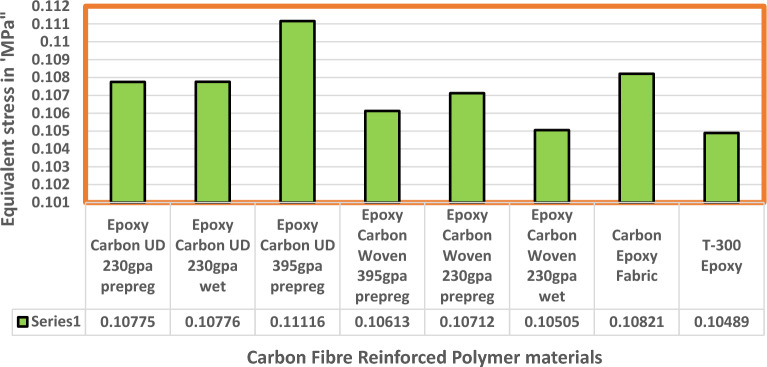
Comparative analysis of Equivalent Stress on Carbon Fibre Reinforced Polymer materials.

Fluid Structure Interaction analysis is mainly focused on determining the best material suitable for the UAV. This analysis was performed for over 20 materials by imposing them to the UAV which include Alloys, composites, plastics, wood, and a few advanced lightweight materials^41–44^. Some of the results are depicted in the Figs. 41 and 42. Results of these comprehensive studies are compared based on five different parameters. They are total deformation of the materials, equivalent elastic strain, strain energy stored by the materials, normal stress acting on them, and finally the equivalent stress acting on the materials. These results are represented graphically in Figs. 43, 44, 45, 46, 47, 48, 49, 50 and 51 in the respective order as mentioned^41–44^.

Based on the results recorded, Titanium alloy, Boron epoxy, CFRP-woven-fabric, and FR-4 based composites are determined as the best performer and is decided that the same four materials will provide better structural integrity for the UAV.Among these titanium alloy came out to be the best performer than all the other materials with a total deformation of 0.00012209 mm which is around 21% lesser than the Boron epoxy which came out to be the second-best performer from the analysis outcomes.This proves that physically the drone when imposed with the suggested material can withstand high amount of aerodynamic loads and also these materials can increase the aerodynamic performance of the drone as they are light righted.

### Control Dynamics

#### Altitude Control of the vehicle

This section introduces a basic concept for the control of the vehicle’s vertical movement. The design of the altitude controller incorporates the translational equation of motion, which establishes the connection between the control input for vertical movement and the acceleration along the z-axis^45–50^.

#### Simplified model

The vehicle dynamics model pertaining to translational motion can be expressed by Eqs. (18), (19), and (20) as documented in references^45–50^.

18 $$\ddot{x} = \left( {\cos \phi \sin \theta \cos \psi + \sin \phi \sin \psi } \right)\frac{U}{m}$$

19 $$\ddot{y} = \left( {\cos \phi \sin \theta \sin \psi - \sin \phi \cos \psi } \right)\frac{U}{m}$$

20 $$\ddot{z} = - g + \left( {\cos \phi \cos \theta } \right)\frac{U}{m}$$

where,$\ddot{x},\,\,\ddot{y},\,\,{\text{and}}\,\,\ddot{z}$ represent the acceleration of the vehicle along the *x*, *y*, and *z* axes, respectively;$\phi ,\,\,\theta ,\,\,{\text{and}}\,\,\psi$ represent the three Euler angles roll, pitch, and yaw, respectively;*m* represents the mass of the vehicle;*g* is the gravitational acceleration; and *U* denotes the control input required to control the vertical movement of the vehicle^45–50^.

The control input *U* directly controls the thrust in the vehicle’s z-axis by controlling angular velocities of the propellers. The control input *U* is given byEquation (21) as follows:

21 $$U = F_{1} + F_{2}$$

where $F_{i} = T_{c} \,\Omega_{i}^{2}$ denotes the thrust force generated by the co-axial propeller *i*; $\Omega_{i}$ is the speed of the propeller *i*; and $T_{c}$ is the thrust constant in N/s^2^.

The Eq. (18) is utilised for the purpose of altitude control. In order to maintain a hovering state, it is necessary to adjust the altitude of the vehicle. During the process of hovering, the vehicle exhibits minimal roll and pitch angles. Hence, the vertical position of the UAV can be mathematically represented by Eq. (22)^45–50^.

22 $$z = \iint {\left( { - g + \frac{U}{m}} \right)}$$

**Figure 52:**
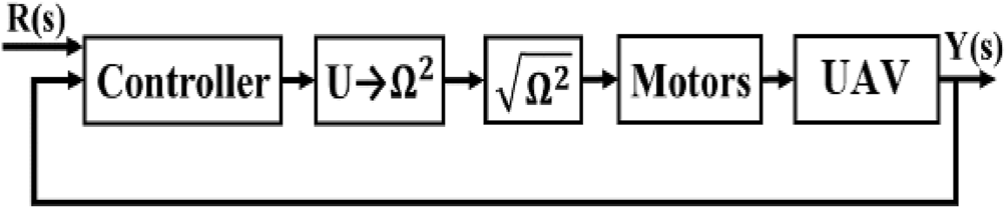
Block diagram of the altitude control system.

The block diagram for the altitude control of the UAV is depicted in Fig. 52. The control input U is generated by the controller based on the reference height and the actual height. This control input is then transformed to squared motor speed by inverting Eq. (19) and afterwards taking the square root. The resulting reference speed is used to directly drive the BLDC motors, which in turn control the vehicle^45–50^.

Where, R(s) represents reference height, Y(s) represents actual height of the UAV.

#### Motor Dynamics

**Figure 53:**
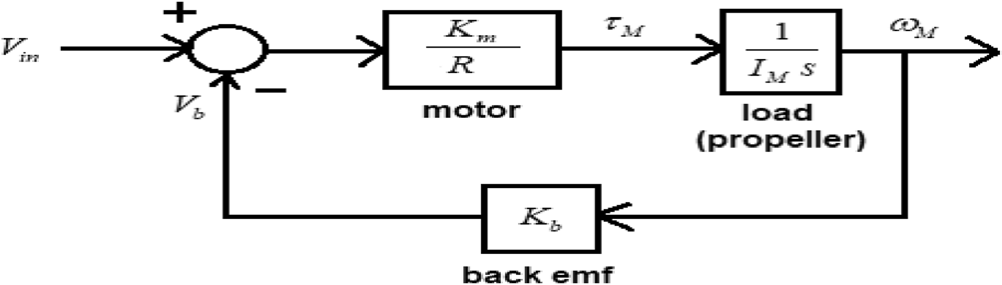
Block diagram of brushless dc motor for single phase with load attached.

The comprehensive control system employed in the altitude control of UAVs should encompass the dynamics of the motors. Typically, motor dynamics are modeled using a first order low-pass filter, where the specific parameter values are determined through empirical experimentation. The mathematical model of the motor is derived based on the electrical and mechanical characteristics of the motor. Figure 53 depicts the block diagram representation of the transfer function model of a DC motor, which includes a load in the form of a propeller.

In Fig. 53, ‘$V_{in}$’ represents the supplied phase voltage, ‘$V_{b}$’ represents the phase voltage back emf, ‘$\tau_{M}$’ represents the torque developed by the motor, ‘$I_{M}$’ represents the moment of inertia of the rotor and the attached propeller, and ‘$\omega_{M}$’ represents the angular velocity of the propeller. The motor used here isREES521600Kv based BLDC. The specifications of the selected DC motor are given in Table 12^45–50^.

**Table 12:** REES521600Kv Brushless Motor BLDC specifications.

Parameter	Value
Motor resistance, *R*	0.09 Ohms
Torque constant,$K_{m}$	0.0059683 Nm/A
Back emf constant,$K_{b}$	0.0059683 Volts/(rad/s)
Rotor inertia, *I* (Including the propeller)	
About x-axis	0.000003788 kg/m^−2^
About y-axis	0.000007459 kg/m^−2^
About z-axis	0.000009604 kg m^−2^

#### Simulink Model of altitude control

**Figure 54:**
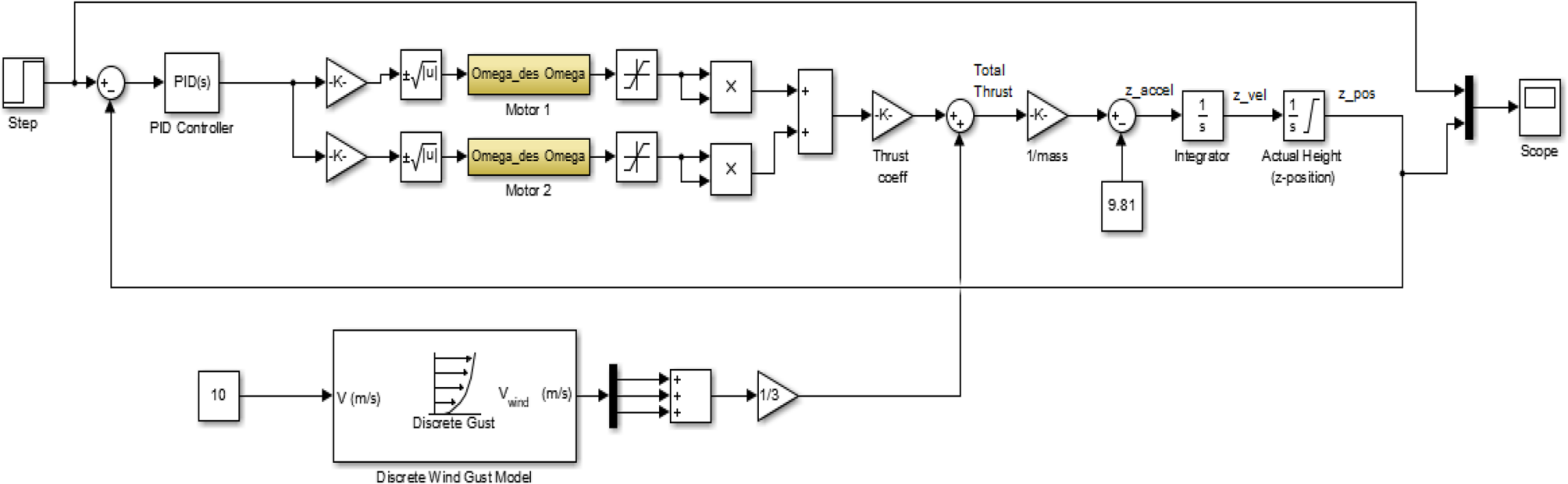
Simulink model of the proposed altitude control system for the UAV.

The Simulink model representing the altitude control system, designed for VTOL and altitude hold, is constructed by integrating the simpler model and the motor dynamics with PID control. The developed model is visually depicted in Fig. 54. The control system incorporates a wind gust model to account for the disturbance input, characterised by a velocity of 10 m/s. To simulate the VTOL reaction, a ramp input is employed, while a step input is utilised to validate the altitude hold response^45–50^.

**Figure 55:**
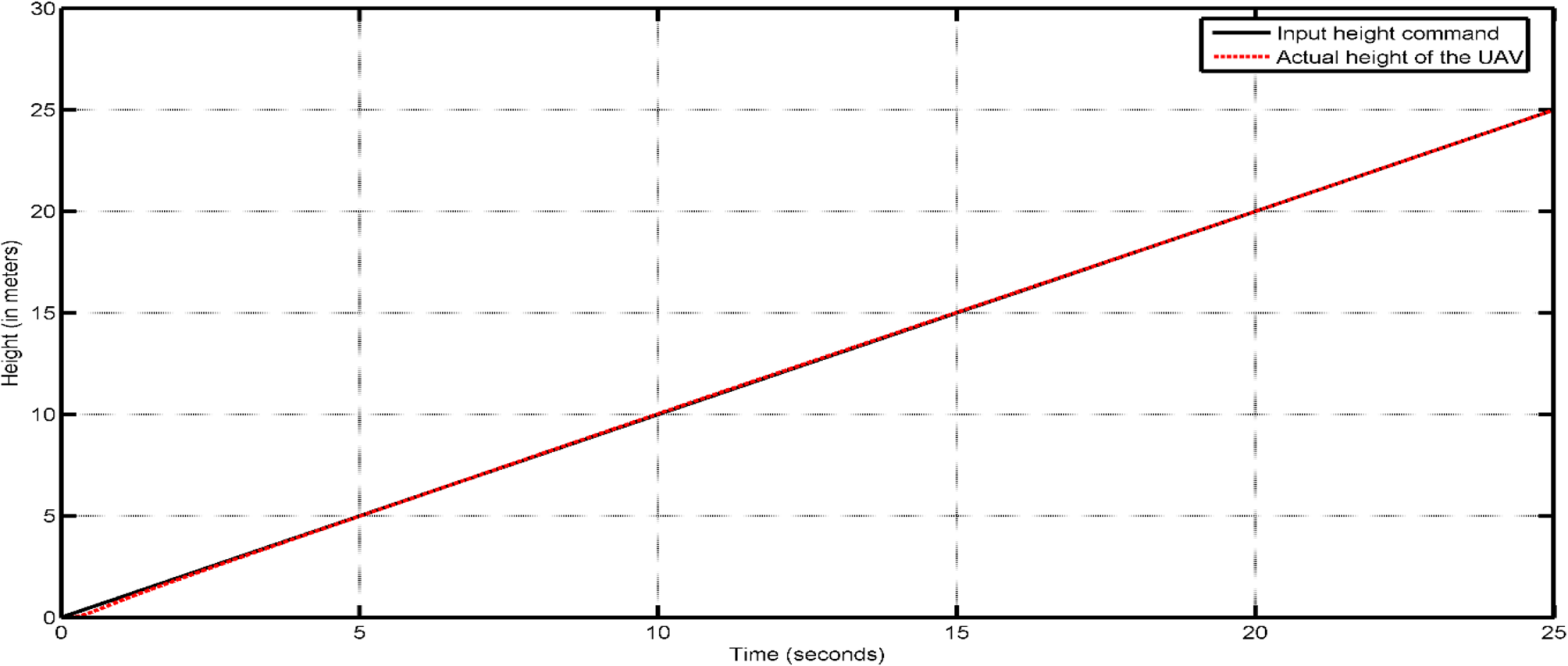
Altitude response of the UAV with tuned PID controller for VTOL operation.

**Figure 56:**
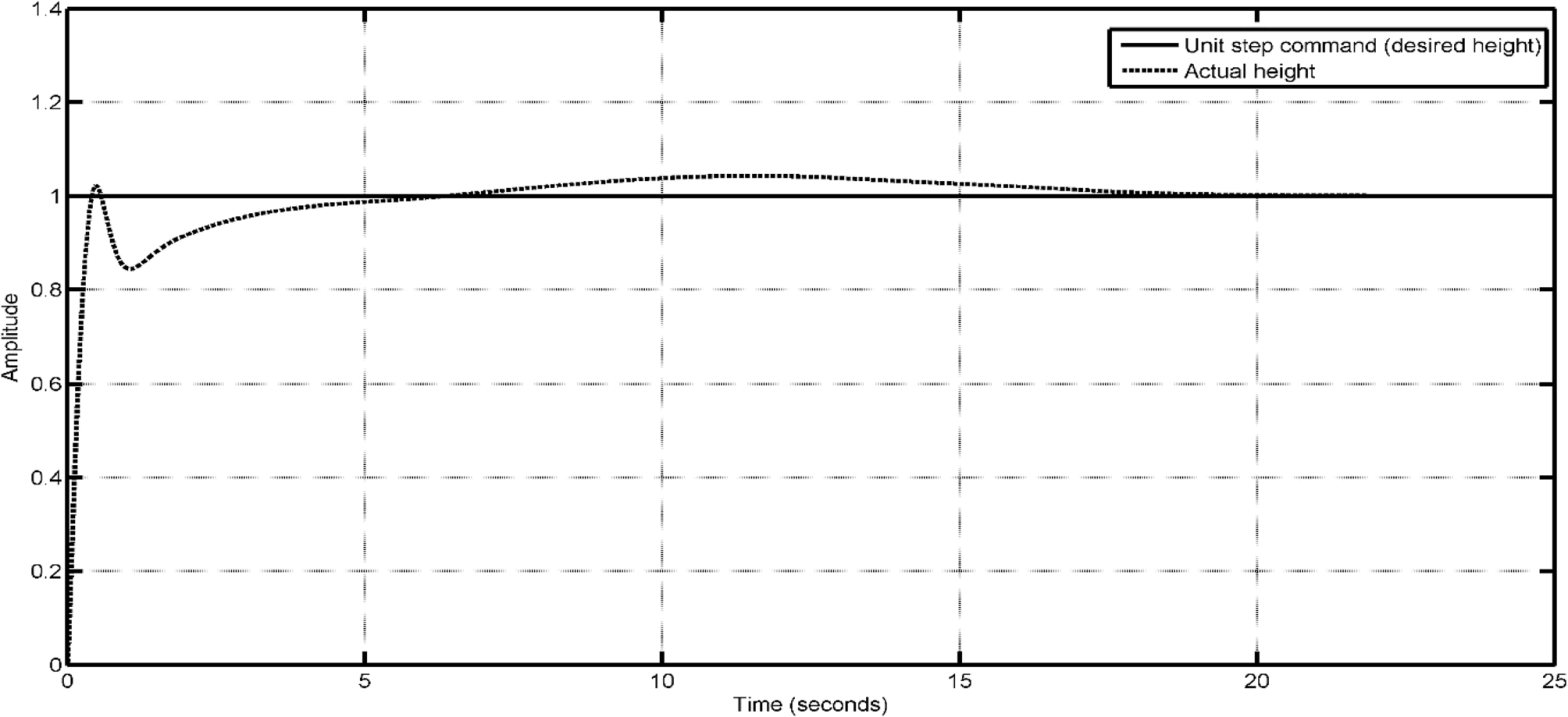
Step response of the UAV with tuned PID controller for altitude hold.

In the Simulink model, some constraints were incorporated to simulate real-world phenomena within our simulation. Initially, the height was constrained by the inclusion of zero as the lowest limit in the integrator responsible for converting z-velocity into z-position, as the UAV is unable to descend beyond the surface of the Earth. The limitation of motor speeds is imposed by saturators, which serve to eliminate negative signals in order to prevent rotation in the opposite direction of the one selected. Certain multi-rotor vehicles include the capability for bidirectional rotor rotation, enabling them to execute aerial flips and sustain inverted flight. However, it is important to note that this aspect is not pertinent to our current discussion. In order to achieve steady operation of the vehicle, a PID controller can be constructed in Simulink by employing straightforward gains. The controller construction of the vehicle involved the utilisation of built-in PID blocks. In this study, the controller gains were adjusted by a manual tuning process employing a straightforward methodology. Table 13 displays the PID gains of the altitude PID controller that has been appropriately calibrated.Fig. 55 provides a more accurate depiction of the vehicle’s actions. In practical aviation scenarios, it is common for a pilot to execute a gradual takeoff during VTOL operations. In order to replicate this phenomenon inside a simulation setting, an altitude command is employed, taking the form of a ramp input. This input signal commences at a value of zero and progressively increases with a constant slope of 1. The seamless take-off and stabilization of the vehicle in response to the gradually increasing altitude command signal can be shown in Fig. 55, with the process completed within duration of 2 s ensures that the drone is capable of performing the actions required in a short period of time. Figure 56 illustrates the operational procedure of altitude hold, wherein a step command is applied to achieve the target altitude even though it spikes up a little as an overshoot, the plot describes that its settling time is very short which makes the drone more stable and easy to control. The UAV exhibits a prompt response to the input, resulting in a 4–5% overshoot before eventually stabilizing at the desired altitude within a time frame of 20 s.

**Table 13:** PID controller gains for altitude control.

Controller	Gain
Proportional	18
Integral	10
Derivative	4

## Conclusions

Surveillance using Hybrid UAV provides a better solution in all aspects as it can be operated from a distance. Normal UAVs can be used but it requires some optimization to make it more efficient to operate at that condition. Hence several optimizations are performed to reach out a better design to meet the requirement. For this, the main design considerations are as follows,

1. Monocopter like configuration i.e., one propulsion system to reduce number of motors used, mainly to reduce weight as motors weigh more than other components.
2. Coaxial power system to reduce tip losses by the propeller thrust and to eliminate the torque produced by single motor.
3. Duct like configuration was placed to increase the velocity of the air produced by propellers.
4. Single rotor maneuvering was attained with the deflectors by producing imbalance at the outlet of the duct.
5. Components of the model will be selected based on the final design through bottom-up approach.
6. The simplified convergent and divergent duct model produced a comparatively higher thrust than the normal ducts.
7. The rate of climb of the drone was significantly increased with the proposed duct configuration.
8. The proposed drone model can reach higher velocities at a lower RPM of the rotors comparatively with the proposed propellers and its coaxial configuration.
9. With the simplified duct configuration, the drag produced very low drag with the minimum amount of skin friction drag.
10. With the coaxial propulsion system and the proposed propellers, the drone was able to perform a stable hovering which is clear from the control dynamics of the drone.

This resulted in the increment of the performance factors of the drone. These factors add on to the advantages for its objective of high-altitude surveillance. For the objective of the surveillance the drone can perform quick altitude climbs with the help of its Coaxial propulsion system and the contribution of thrust from the duct, and it also has the ability to be stable and to return to its steady state even after disturbances in a short period of time which is a key factor for high altitude surveillance. This advantage of the Drone was verified during the control dynamics analysis.

One of the takeaways from this research is that conventional C–D ducts will be able to produce a high exit velocity but the structural into of the duct will be very low compared to the proposed duct model.

Initially the duct design 1 analyzed, produced five times more thrust at the outlet of the divergence section. Hence, the project proceeded with the design. The addition of other design parts like the fuselage, propeller, and deflectors were for the successive model designed. The addition of the various components reduced the velocity that we attain in duct alone model. Another duct design was also considered for better optimization for the design to meet the requirements. Based on the analysis, Duct design 2 is effective overall as it produces higher velocity comparatively over 19% higher velocity than the other and low pressure for same inlet velocity with 4% lesser pressure acting on it. In the case of Deflectors, the moment required to attain directional changes are obtained as expected. From the results, the required amount of moment is obtained at a low angle i.e., 15 degrees a data higher angle, drag is higher than the lift hence it can be used in some other operating conditions. From the outcomes the deflector plates attached to the base of Duct 2 produced over 30% more lift than the other to create higher moments. In the case of VTOL and cruising, we can see a negligible decrease in outlet velocity which is due to incorporation of other components. While considering pressure changes in all steady flow cases there is no such contribution to the downfall of the UAV. Hence, as an overall, Design 2 is observed as a better model when compared. The experimental validation results are approximately the same as the theoretical and numerical analysis. Then from the outcomes of the structural analysis, it was determined that titanium alloy was the best performing material among other materials which include composite materials such as glass fibres and carbon fibres with a total deformation of 0.00012209 mm which is 21% lesser than Boron epoxy composite, the second best performing material having a deformation of 0.00014802 mm. The dynamic control changes that were simulated using MATLAB Simulink show its response while hovering and the response when the throttle is increased. And also, the response of the UAV when there is a gust load. The simulations show that the drone is capable of stabilizing itself in a short period of time even after the presence of some overshoot. It validates the components that were selected.

## Data Availability

The authors confirm that the data supporting the findings of this study are available within the article.
